# UNMASC: tumor-only variant calling with unmatched normal controls

**DOI:** 10.1093/narcan/zcab040

**Published:** 2021-10-06

**Authors:** Paul Little, Heejoon Jo, Alan Hoyle, Angela Mazul, Xiaobei Zhao, Ashley H Salazar, Douglas Farquhar, Siddharth Sheth, Maheer Masood, Michele C Hayward, Joel S Parker, Katherine A Hoadley, Jose Zevallos, D Neil Hayes

**Affiliations:** Public Health Sciences, Fred Hutchinson Cancer Research Center, 1100 Fairview Ave N, Seattle, WA 98109, USA; Lineberger Comprehensive Cancer Center, University of North Carolina at Chapel Hill, 101 Manning Drive Chapel Hill, NC 27514, USA; Center for Cancer Research, University of Tennessee Health Science Center, 19 South Manassas, Memphis, TN 38163, USA; Lineberger Comprehensive Cancer Center, University of North Carolina at Chapel Hill, 101 Manning Drive Chapel Hill, NC 27514, USA; Otolaryngology Head and Neck Surgery, Washington University School of Medicine, 660 South Euclid Avenue, Campus Box 8115, St. Louis, MO 63110, USA; Center for Cancer Research, University of Tennessee Health Science Center, 19 South Manassas, Memphis, TN 38163, USA; Lineberger Comprehensive Cancer Center, University of North Carolina at Chapel Hill, 101 Manning Drive Chapel Hill, NC 27514, USA; Lineberger Comprehensive Cancer Center, University of North Carolina at Chapel Hill, 101 Manning Drive Chapel Hill, NC 27514, USA; Lineberger Comprehensive Cancer Center, University of North Carolina at Chapel Hill, 101 Manning Drive Chapel Hill, NC 27514, USA; Otolaryngology, University of North Carolina at Chapel Hill School of Medicine, 101 Manning Drive Chapel Hill, NC 27514, USA; Lineberger Comprehensive Cancer Center, University of North Carolina at Chapel Hill, 101 Manning Drive Chapel Hill, NC 27514, USA; Oncology, University of North Carolina at Chapel Hill School of Medicine, 101 Manning Drive Chapel Hill, NC 27514, USA; Otolaryngology, University of Kansas Medical Center, 3901 Rainbow Blvd, Kansas City, KS 66160, USA; Lineberger Comprehensive Cancer Center, University of North Carolina at Chapel Hill, 101 Manning Drive Chapel Hill, NC 27514, USA; Lineberger Comprehensive Cancer Center, University of North Carolina at Chapel Hill, 101 Manning Drive Chapel Hill, NC 27514, USA; Genetics, University of North Carolina at Chapel Hill School of Medicine, 101 Manning Drive Chapel Hill, NC 27514, USA; Lineberger Comprehensive Cancer Center, University of North Carolina at Chapel Hill, 101 Manning Drive Chapel Hill, NC 27514, USA; Genetics, University of North Carolina at Chapel Hill School of Medicine, 101 Manning Drive Chapel Hill, NC 27514, USA; Otolaryngology Head and Neck Surgery, Washington University School of Medicine, 660 South Euclid Avenue, Campus Box 8115, St. Louis, MO 63110, USA; Center for Cancer Research, University of Tennessee Health Science Center, 19 South Manassas, Memphis, TN 38163, USA; Lineberger Comprehensive Cancer Center, University of North Carolina at Chapel Hill, 101 Manning Drive Chapel Hill, NC 27514, USA; Internal Medicine, Division of Hematology-Oncology, University of Tennessee Health Science Center, 19 South Manassas, Memphis, TN 38103, USA

## Abstract

Despite years of progress, mutation detection in cancer samples continues to require significant manual review as a final step. Expert review is particularly challenging in cases where tumors are sequenced without matched normal control DNA. Attempts have been made to call somatic point mutations without a matched normal sample by removing well-known germline variants, utilizing unmatched normal controls, and constructing decision rules to classify sequencing errors and private germline variants. With budgetary constraints related to computational and sequencing costs, finding the appropriate number of controls is a crucial step to identifying somatic variants. Our approach utilizes public databases for canonical somatic variants as well as germline variants and leverages information gathered about nearby positions in the normal controls. Drawing from our cohort of targeted capture panel sequencing of tumor and normal samples with varying tumortypes and demographics, these served as a benchmark for our tumor-only variant calling pipeline to observe the relationship between our ability to correctly classify variants against a number of unmatched normals. With our benchmarked samples, approximately ten normal controls were needed to maintain 94% sensitivity, 99% specificity and 76% positive predictive value, far outperforming comparable methods. Our approach, called UNMASC, also serves as a supplement to traditional tumor with matched normal variant calling workflows and can potentially extend to other concerns arising from analyzing next generation sequencing data.

## INTRODUCTION

Although variant detection and mutation calling within next-generation sequencing (NGS) data have been the source of much investigation, gaps and shortcomings remain. Most methodological development has been in the areas of detection of population variants or somatic mutations in the setting of matched tumor-normal (MTN) samples. Importantly, neither of these two approaches represent the most common clinical application in which clinicians attempt to characterize disease variants, often from an abnormal cancer genome without a matched reference sample.

While common germline variants (GVs) can be identified with germline databases (GDs) such as dbSNP (1), 1000 Genomes (2) and ExAC (3), rare GVs require more sophisticated methods to identify. The fundamental approach involves identifying genomic intervals of GV calls at similar B allele frequencies (BAF) and then inferring the germline status of a variant, using their variant allele frequency (VAF), through some notion of ‘distance’ or posterior probability. A subset of existing methods (4–6) follow this basic procedure to identify subject-specific or rare GVs. One unavoidable limitation of all methods is distinguishing GVs from somatic for highly purified samples or cell lines since the BAF and VAF are likely to overlap for variants that initiate oncogenesis (founder mutations) and present on all copies of a haplotype (within cancer cells, thus appearing germline for a purified sample). LumosVar (6) demonstrated this limitation through simulation.

In this setting, the vast majority of patients do not submit material for MTN sequencing, and published methods in this area are generally lacking. In this report we describe a workflow and software, Unmatched Normals and Mutant Allele Status Characterization (UNMASC) that applies and adapts a series of best-practices techniques for the purpose of highly sensitive, specific, and precise variant detection.

In its simplest form, somatic mutation detection is similar to genotyping, or the detection of non-reference bases within NGS data. Unlike genotyping, mutation calling generally considers as uninformative (i.e. not disease-causing) the vast majority of variants that are present in the germline of the individual. Improved classification of variants as germline versus somatic in diseased tissue without matched normal (MN) samples, while at the same time excluding other variants as likely sequencing errors or alignment errors is an area of unmet need. GVs can be identified by several methods including the use of a MN control, GDs, or inferred by the sequencing process or through modeling. In the current manuscript, we will consider the strengths and weaknesses of each of these approaches while considering other sources of artifactual variants in NGS data.

Classification is the main objective of tumor-only (TO) variant calling. Models designed to perform classification require a sufficient sample size (pooling both samples and variants) and reliable labels (e.g. somatic, germline, other). The presence of artificial variants (AVs) in general can hinder classification performance. Studies have demonstrated that AVs arise from various steps in a sample’s processing (7–15). Therefore at the very least, when constructing a training dataset for variant classification, the labels should include germline, somatic and various classes of AVs. Without accounting for AVs, methods that train models to classify variants as only germline or somatic will be at a disadvantage. In addition, constructing a training dataset composed of MTN variant calls assumes that all underlying somatic variants (SVs) were captured and that each tumor’s MN served as a reliable control. AVs, such as oxoG (10), strand bias (9), and paraffin-induced (15–17), present among MTN variants led us to avoid comparisons with supervised methods that trained models on only germline or somatic variant classifications. Also, existing unsupervised methods that perform tumor copy number segmentation based only on variants reported in GDs run the risk of being negatively impacted by clusters of AVs presenting in GDs. This can be seen with low VAF calls in GDs that do not present in the underlying MN.

### UNMASC overview

UNMASC improves on concepts proposed by Hiltemann et al. (18), MuTect (19) and LumosVar (6) in which pools of unmatched normals (UMNs) improve or replace a MN for the purpose of variant detection. We document the role of pooled normal in quantification of a variety of sequencing and alignment AVs and apply a series of locus and sample-specific filters and annotations. A major drawback of current variant filtering methods (4–6,18,20–24) is the pre-filtering of variants that can provide crucial genomic context for understanding the remaining prioritized variants. With this in mind, UNMASC purposely utilizes the nature of subsets of variants that reveal local or genome-wide germline and multiple forms of AV clusters. Most steps of UNMASC’s variant annotation involves applying a criteria to subset variants, summarizing the subsetted variants by proposed parametric distributions and then quantifying the relationship between each variant and the overall distribution of subsetted variants. To further strengthen our approach and provide novelty, utilizing UMNs provides fundamental locus-specific and regional genomic context in terms of variant calling with respect to the reference genome and bioinformatic workflow. These steps provide the user with data-driven annotations.

UNMASC integrates public database annotations, sequencing metrics and data-driven annotations to retain variants meeting variant quality score, germline population allele frequency, read depth, strand bias *P*-value, predicted AV frequencies, Cosmic count, variant prediction and non-germline posterior probability thresholds. Details associated with UNMASC’s filtering criteria are presented in Supplemental Materials Section S4.9. Depending on the user’s intentions, strict or liberal variant filtering can be applied for which we offer empiric guidance based on a large benchmarking cohort.

## MATERIALS AND METHODS

### Data background

For the purpose of assessing our ability to detect and characterize somatic variants in cancer, we assembled a cohort of patients with clinically validated mutations from a clinical trial of next-generation sequencing in cancer using a target panel called UNCseq™ (LCCC1108: NCT01457196). From a total cohort of 1500+ patients, we selected a representative subset at random of 100 patients with a spectrum of clinical and genomic parameters relevant to a robust method. All patients had paired tumor and normal samples to benchmark as well as clinical confirmation of relevant clinically actionable mutations (25,26).

To assess the performance of our pipeline in the setting of UMNs, a set of 20 UMN samples independent of the tumor set was selected from the larger UNCseq™ cohort. The determination that 20 samples would be sufficient was empiric but ultimately proved to be a greater number than was necessary for our purposes. A summary of tumor and normal benchmark sample demographics is provided in Table 1.

**Table 1.** Summary demographics of the 100 tumor and 20 LCCC1108 UMNs. Sequencing metrics include median and range statistics on percent bases with ≥100× coverage, average number of reads covering target regions and pathologist-derived tumor percent

Variable	Normal	Tumor
	*N*(%)	*N*(%)
Gender		
Female	16(80.0%)	75(75.0%)
Male	4(20.0%)	25(25.0%)
Race		
Black	6(30.0%)	16(16.0%)
Other	1(5.0%)	7(7.0%)
White	13(65.0%)	77(77.0%)
Age at diagnosis		
20 to 52	4(20.0%)	36(36.0%)
53 to 62	7(35.0%)	33(33.0%)
63 to 82	9(45.0%)	31(31.0%)
# Confirmed mutations		
1	6(30.0%)	36(36.0%)
2	8(40.0%)	26(26.0%)
3	3(15.0%)	20(20.0%)
4+	3(15.0%)	18(18.0%)
	Median(range)	Median(range)
Target bases with 100× (%)	97.6 (71.7–98.6)	97.5 (76.9–99.2)
Mean target coverage	818.9 (155.8–1190.2)	831.6 (175.7–2048.1)
Percent tumor*	65 (30–90)	70 (20-90)

*Percent tumor in benchmark tumor samples and corresponding tumor samples for UMNs.

Benchmark tumor samples consisted of brain/central nervous system, breast, gastrointestinal, genitourinary, gynecologic, head and neck, hematologic, lung, lymphoma and musculo-skeletal tumor types for the UNCseq™ cohort.

### Position filtering

**Figure 1.**
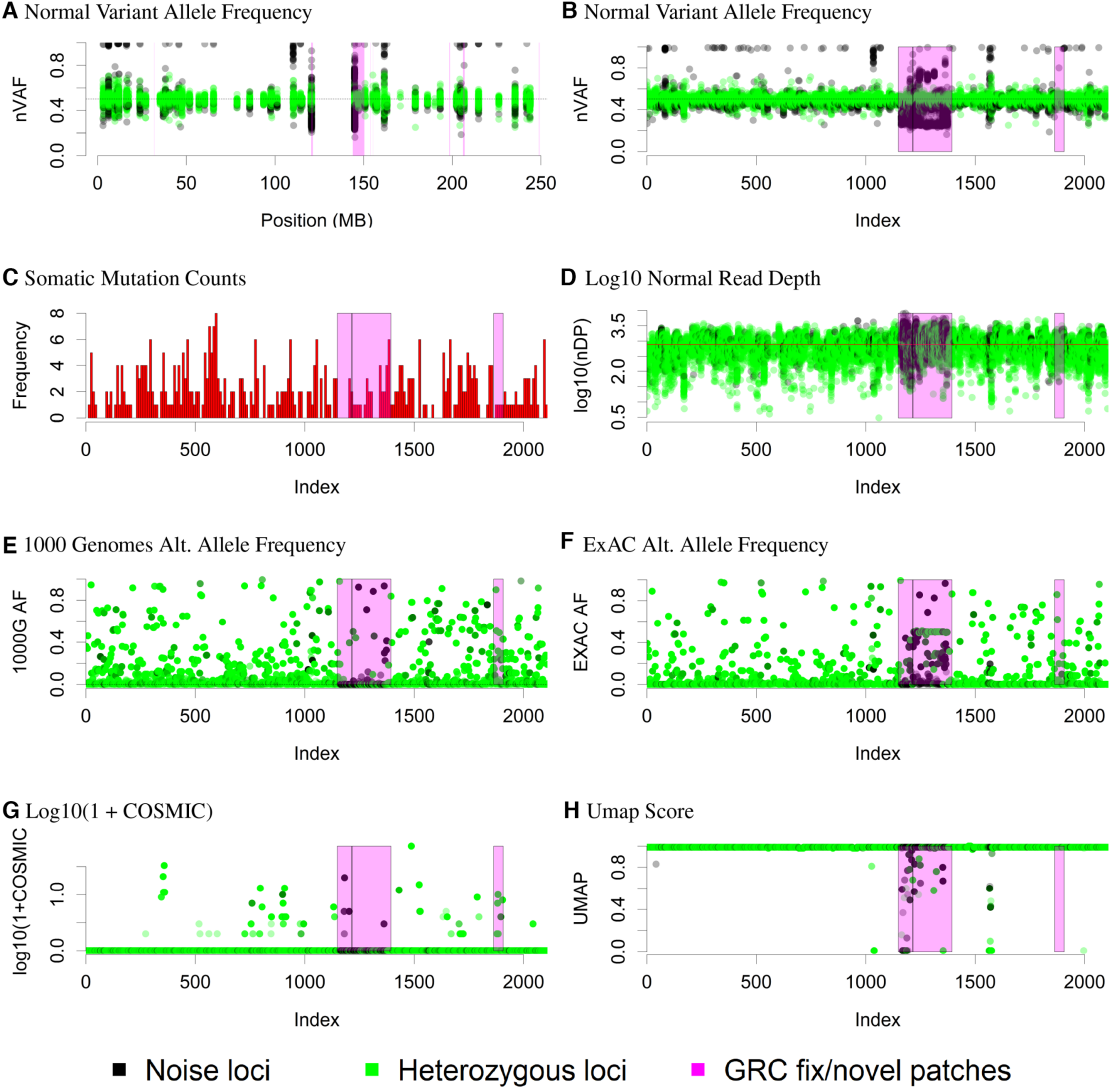
Chromosome 1 visualization. Normal sample loci classified as either noise or heterozygous. Figures (**A**) and (**B**) plot the normal nVAF on the *y*-axes. While the *x*-axis denotes the genomic position in megabases for Figure (A), the *x*-axes for Figures (B–H) denote the ordered genomic loci of positions. Figure (**C**) illustrates the distribution of loci where somatic variants were called. In Figure (**D**), the *y*-axis denotes the log10 of total read depth of clustered normal read counts with the line indicating the median. In Figures (**E–H**), the *y*-axes denote the 1000 Genomes population allele frequencies (AF), ExAC population AF, log10 of 1 + COSMIC counts, and Umap scores, respectively.

While our work is aimed at somatic mutations in tumors, we wanted to begin by observing how alternate and reference bases present in normal samples. All samples were processed with a hybrid capture method however this analysis can be applied to whole genome and whole exome sequencing too. Since one of the most useful characteristics of variants is their allele frequency (AF), we looked at AFs in the normal samples without regard to tumor, focusing on positions that were not called homozygous. The hypothesis was that at least some non-reference variants in MN from tumor samples would demonstrate AFs other than a Mendelian distribution of 50% or 100%. Such positions would be problematic since they could be falsely interpreted as somatic alterations in regions of copy number gains or losses when detected in tumor cases. We further interrogated the variant position in association with existing public database annotations. For the 100 tumors, Isaac (27) was run on each of their corresponding normal BAM files to generate normal genome VCFs. For each sample, loci located within targeted gene panel capture regions were retained and variants were clustered based on VAF (Supplementary Materials Section S4.2). Loci with nVAF classified near to zero (primarily sequencing errors) and one (homozygous) were excluded based on clustering assignment. Remaining loci from the 100 normal samples were pooled together for visualization in Figure 1 and hypothesis testing in Table 2.

**Table 2.** The cross tabulation of counts between variables defined by genomic annotations and the outcome are presented

			Univariate	Multivariate
Variable	Label		OR 95% CI (*P*)	OR 95% CI (*P*)
	Het.	Noise		
Umap			0.68–0.85 (1.85e-06)	0.69–0.87 (1.33e-05)
NO	1034	10840		
YES	539	4320		
GRC			3.22–4.11 (8.60e-107)	3.04–3.90 (1.21e-84)
NO	11377	497		
YES	4193	666		
GS			1.43–1.82 (2.46e-15)	1.17–1.51 (9.18e-06)
NO	11115	759		
YES	4376	483		
SV			3.87–4.92 (5.53e-148)	3.65–4.66 (5.62e-115)
NO	11388	486		
YES	4096	763		

Umap = base uniqueness score is ≥0.99. GRC = base is contained within a fix/novel patch. GS = base is present in a GDs (ExAC or 1000 Genomes) and COSMIC database. SV = a somatic variant was called within 10 bases of the base considered. Univariate and multivariate analyses are presented in terms of odds ratios (OR), 95% confidence intervals (95% CI), and *P*-values (*P*).

As expected, the bulk of all remaining positions centered around nVAF of 0.5. Clustering then provided an objective means of assigning a variant to the heterozygous state plus or minus measurement error versus the alternative (neither 0, 0.5 or 1) (Figure 1). Samples in the alternative state were considered as non-Mendelian or simply ‘noise’. Difficult to genotype regions have been characterized previously by efforts such as Umap (8) (Figure 1) and often noted in regions of lower genome complexity such as near centromeres. The challenge posed by these regions might initially appear trivial since the genomic territory involved covers a small fraction of the genome. However, the scale becomes more daunting when the results are indexed by variant order rather than position (Figure 1B), demonstrating that these narrow regions generate large numbers of variants. Closer examination reveals that in genomic intervals of unexpected nVAF deviations (such as the chr1 centromere), only excluding loci with decreases in Umap score was too conservative. Several loci classified as Mendelian corresponded to decreases in Umap and other loci with Umap scores near 1.0 were classified as non-Mendelian suggesting that Umap alone is insufficient for identification of these challenging genome positions.

We then considered if these non-Mendelian germline positions were associated with somatic mutation calls in cancer databases. Such variants would likely be false positives since they violate the assumptions of many variant detection algorithms. We confirmed that many variants were reported in these challenging regions (Figure 1C). We further excluded sequencing depth as a meaningful contribution to variant allele imbalance (Figure 1D). Given that we were unaware of existing methods to consider allele imbalance in this manner, we interrogated the AFs in our sample set of variants previously reported in public datasets including 1000 Genomes (2), ExAC (3) and COSMIC (28). Importantly, for normal sample non-Mendelian variants in the UNCseq™ cohort and also reported in 1000 Genomes, these loci had low population AF suggesting underlying AVs rather than rare population variants (Figure 1E). Variants overlapping with ExAC were more likely to have ExAC AFs of 0.5 but still with many ExAC AFs divergent from 0.5 (Figure 1F). Finally we observed that many of these difficult positions were present in the COSMIC database as somatic variants, concerning for false positives. We also overlay genomic interval novel/fix patch information with version GRCh37.p13 provided by the Genome Reference Consortium (GRC) (12) as a possible cause of the non-Mendelian AF distributions. In order to formally quantify the patterns seen in Figure 1 across all genome positions and all samples, we modeled the probability that a locus was ever classified as non-Mendelian as a function of being located within a GRC patch, Umap score ≥ 0.99, presenting in both germline and somatic databases, and finally if a somatic variant was called within 10 bases of the locus (Table 2). Our analyses revealed strong associations between each of the four covariates and non-Mendelian status, suggesting our initial classification procedure aligned with existing genomic annotations. While direct interpretation of such positions might be complex, this locus classification provides the user additional insight into a variant that might otherwise be considered clinically meaningful when it is annotated as non-Mendelian among normal controls. In other words, variants called in this position have higher probability of being false positives. We then considered the multivariate model. For model predictiveness, the AUC of the multivariate model is 0.610 with a McFadden R-squared of 0.051. These results suggested that existing variant annotation such as Umap for challenging regions alone was highly statistically associated with non-Mendelian status but fails to account for most false positive variant calls. The limited prediction can be attributed to how the outcome variable was naively characterized. For example, within a given GRC patch, there was a mixture of variants near normal VAF of 0.5 as well as variants deviating from 0.5. Our analyses suggested GRC patches were more associated with increased deviations but would have difficulty inferring the status of variants as likely true versus likely false within a GRC patch. The other three covariates appeared informative of nVAF deviations from 0.5 in a subset of regions but did not capture overall regions of deviation. Chromosomes 1 and 6 (Supplementary Figure S1) have regions in which GRC regions correlated with our observation of abnormal VAF. In other cases, however, there were regions in which we detected abnormal VAF not in association with a GRC region, and we observed this along chromosomes 7, 14, 16 and 19 (Supplementary Figures S2–S5, Supplementary Section S2). Motivated by these results, we developed an empiric measure of baseline allelic fraction noise across the genome referred to as hard to map (H2M) regions (Supplementary Materials Section S4.3). A simulated example of H2M regions is also provided (Supplementary Figure S7).

#### H2M application to tumor only variant detection

Having shown that H2M positions have unfavorable properties for variant detection in normal samples, we conclude that these positions are even more unfavorable for tumor variant detection. To address this challenge, we considered different approaches to identify and penalize such positions. Importantly, we demonstrated that such positions can be empirically determined from non-cancer controls characterized by the sequencing platform configuration (such as capture bait set, sequencing strategy such as depth or read length) as opposed to being inherent properties of the genome itself. Accordingly, rather than creating a catalog of all H2M regions, we assess each genome positions on the fly on a sample by sample basis through nVAF segmentation applied to each tumor benchmark sample across all detected variants. In this novel approach UNMASC pools nVAFs from UMNs to identify gene regions specifically challenging for variant calling. Interestingly, these H2M regions have variable impact on variant detection across samples and loci. For example, variants detected in genomic intervals in the genes *PDE4DIP* and *NOTCH2* were deemed H2M in all benchmark samples. By contrast, *ANTXR1*-contained variants were called in H2M regions among 22 of the 100 tumors. Our approach is not the first to identify genes with challenges to variant calling. For example, authors of SGZ (5) acknowledged that H2M genomic regions exist but their algorithm addresses this at the gene level rather than at the problematic DNA sequence. For example, SGZ excluded all variants along *HLA* and *CYP2D6* genes, whereas we will show that UNMASC is able to identify H2M regions within *HLA-A*, *HLA-B*, *CYP2D6* and others without resorting to exclude all variants along each gene (Supplementary Table S3).

## RESULTS

### Benchmark variant samples and somatic mutations

Samples were sequenced using hybrid capture technology and Illumina paired-end sequencing technology on the HiSeq2000/2500 and NextSeq500 machines as has been reported elsewhere (25). For the purposes of developing a gold standard set of variants, we compared those variants obtained when a matched tumor normal pipeline was utilized versus the results obtained when the same samples where assessed compared to a tumor-only pipeline utilizing non-matched reference controls. Briefly, variants were called and combined using Strelka (29), UNCeqR (30) and Cadabra (31) and annotated with Oncotator (32). As documented above, variants in H2M regions are problematic even when MNs are available. As such, variants present in MTN calls were excluded from the gold standard set if they were located in H2M intervals unless they presented in COSMIC with at least 10 counts.

To generate the experimental set of variants, the pipeline was run for each tumor against each of 20 normal controls. The variants from all 20 runs were collected and passed into UNMASC’s workflow described below. The outcome of interest in the study was whether or not a variant detected by the paired MTN analysis was also detected by the UNMASC tumor only pipeline. A variant was assessed to be present at a position if it was detected by consensus across the iterated control runs. We confirmed experimentally that 100% detection of a variant across all normal controls was suboptimal because variable coverage and other factors were responsible for occasional false negative variant detection in any individual normal control at a specific location. At thresholds of 25%, 50%, 75%, 90% and 100% across all 100 samples led us to select 90% as an appropriate threshold for the proportion of UMNs a variant was called in. This threshold provided a variant call with robustness since 100% was too stringent for some samples when at least one UMN failed to call a variant. On the other hand, a threshold <90% led to highly variable PPV (due to random draws of UMNs) for some samples. The pipeline’s performance was assessed by sensitivity (SENS) and positive predictive value (PPV) with the gold standard filtered MTN variants described above as the test statistics. As a secondary validation we interrogated a subset of all variants that were manually reviewed and clinically validated by secondary clinical lab testing in the clinical trial.

#### UNMASC workflow

**Figure 2.**
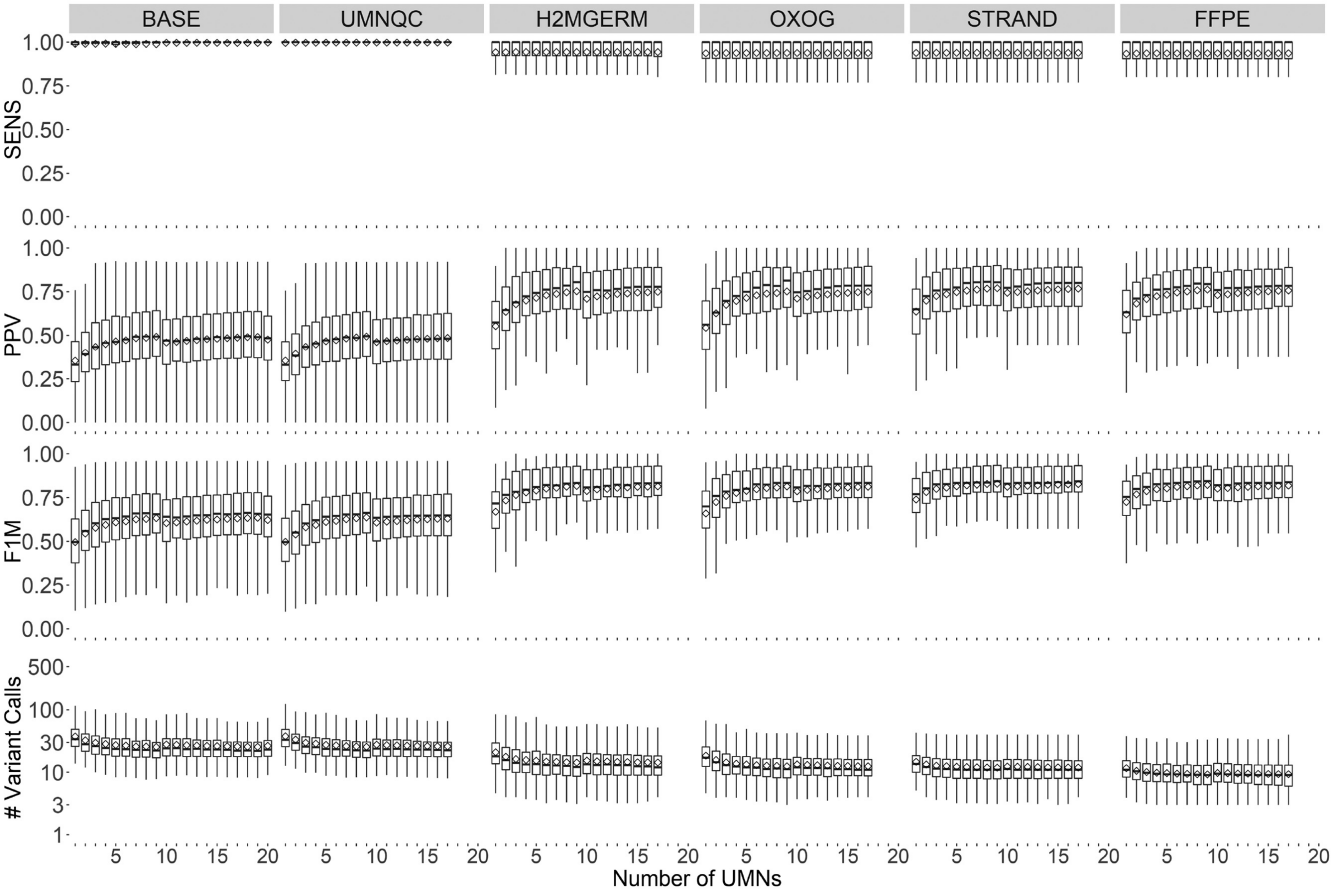
Overall performance across the 100 benchmark tumor samples. Box plots were generated by averaging within each subject’s performance metrics for the 90% frequency threshold required to call a variant somatic. Columns correspond to the six cumulative filtering criterion (BASE includes depth, Qscore, targeted capture, low/moderate/high impact, ExAC, 1000 Genomes filtering, UMNQC includes BASE and excluding low quality UMNs, H2MGERM includes UMNQC along with H2M and GVs filtering, OXOG includes H2MGERM and removing oxoG variants, STRAND includes OXOG along with removing strand bias AVs, and FFPE not only includes STRAND but also excludes paraffin AVs). Rows correspond to sensitivity (SENS), positive predictive value (PPV), F1 measure (F1M) and number of filtered variant calls (#fVCs). The *x*-axis denotes the number of randomly selected UMNs to perform the variant calling. SENS, PPV, F1M, #fVCs were averaged among the 10 instances of UMN draws at the 90% UMN call threshold within a sample and summarized by boxplots.

To obtain a high sensitivity, it is necessary that the initial variant calling liberally collects the sum of all possible variants. Filtering of variants from the initial set was hypothesized to improve the specificity and PPV. We therefore considered the following filtering steps as part of the UNMASC workflow (Figure 2 and Supplementary Section S4). In keeping with convention, we first defined a concept of sequencing quality filtering criteria ‘BASE’ corresponding to filtering variants on read depth (≥10), Qscore (≥10), targeted capture region, retaining low/moderate/high impact exonic variants and present below a specific population allele frequency in ExAC and 1000 Genomes (excluding common SNPs) (Supplementary Section S4.7). We defined a second criteria ‘UMNQC’ (UMNs quality control) which improves UNMASC by recognizing that lower quality normal controls negatively impact SENS and PPV and should be removed. Criteria ‘H2MGERM’ removes penalized variants from H2M regions and variants in germline equilibrium (VIGE). VIGE variants are those variants which appear to have the same allele fraction as known SNPs in their shared clustered region but are not present in a SNP database above a prespecified population threshold. Such variants are most likely private germline SNPs but could represent at least two important alternative classes: founder driver gene mutations which occur early in tumorigenesis and somatic mutations in samples with very high tumor purity. VIGE variants with posterior probability >0.5 are excluded but the user might wish to interrogate these specifically for the possibility of highly pure tumors or founder mutations (Supplementary Materials Section S4.6). A fourth criteria ‘OXOG’ penalizes and excludes positions as likely due to the oxoG artifact (loci clustered to the tumor VAF cluster characterized by base substitutions C>A/G>T, Supplementary Materials Section S4.5). The fifth criteria ‘STRAND’ penalizes and excludes strand bias variants (Fisher test *P*-value <0.05, Supplementary Materials Section S4.4). Lastly, the criteria ‘FFPE’ penalizes and excludes identified paraffin variants (loci clustered to the tumor VAF cluster characterized by base substitutions C>T/G>A and indels). In the current implementation the strand bias does not consider context of the trinucleotide sequence but future implementations might. Details of UNMASC’s overview workflow are provided in the Supplementary Materials Section S4.8 and 4.9 and depicted in Supplementary Figure S8 along with a table summary in Supplementary Table S2.

We evaluated UNMASC’s performance across the benchmark samples and the filtering criteria (Figure 2). Considering only quality filtering approaches (BASE criteria) generated overall very high sensitivity, which was reassuring, but disappointingly low PPV and an overall large number of variants per samples. By considering optimal number and nature of normal controls (UMNQC criteria) we observed that the sensitivity approached 100%, no matter which normal was used and no matter how many were employed. However, we clearly demonstrated that a subset (*n* = 3) of UMNs demonstrated unfavorable properties and their exclusion improved the results overall (Figure 2, column BASE). The impact of each filtering criteria can also be observed for one selected sample (Supplementary Section S5 and Supplementary Figure S10). We determined that compared to other controls, the unfavorable controls demonstrated clustering in sequencing quality metrics toward the lower end although they remained in the acceptable range (Supplementary Figure S6 and Supplementary Section S3).

As part of the selection of normal controls, we considered the impact of race as a measure of genetic diversity in the selection of controls. Racially diverse controls resulted in higher numbers of variants detected and they did not decrease the sensitivity. Additionally, although racially mismatched controls generated higher numbers of variants, these did not generally inflate false positives because they were filtered out of the combined variant set which required a variant to occur in 90% of all tumor-normal pairs. Removing the three under-performing UMNs from the dataset increased the average sensitivity by a small amount, by removing outlier cases of lower sensitivity (Figure 2, column UMNQC). Although sensitivity was high overall after considering the BASE and UMNQC criteria, the total number of variants per assay remained high (*n* = 50 on average for 3 mb of genome interrogated) and the PPV was modest overall (50%).

We observed that increasing numbers of normal controls per sample decreased the false positive calls as expected by increasing the chances that any single normal control would contain a rare SNP that might otherwise be called a mutation. Somewhat unexpectedly, this benefit was marginal and plateaued at around 10 normal controls. We then considered the impact of removing both variants in H2M regions (variants within a genomic segment where the majority of nVAFs harboring a mixture of reference and non-reference reads deviate from 0.5) and VIGE. The H2M critera produced a significant decrease in the total number of variants called and a significant increase in the PPV (Figure 2, column H2MGERM) with overall little impact on sensitivity. Examples of wide and narrow regions consistently identified as H2M are presented in Supplemental Figure S9.

**Figure 3.**
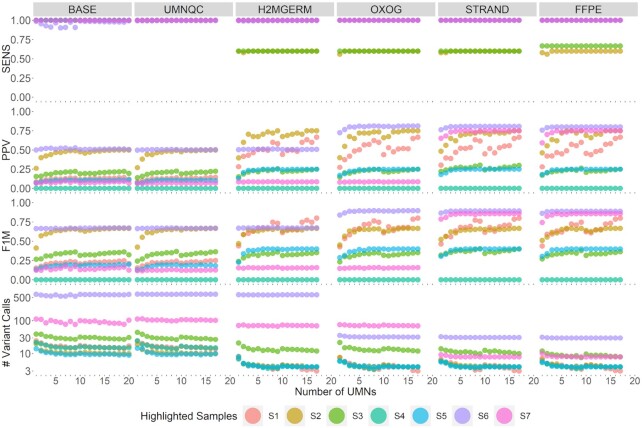
Performance of seven highlighted tumor samples. Columns correspond to the six cumulative filtering criterion (BASE includes depth, Qscore, targeted capture, low/moderate/high impact, ExAC, 1000 Genomes filtering, UMNQC includes BASE and excluding low quality UMNs, H2MGERM includes UMNQC along with H2M and germline variant filtering, OXOG includes H2MGERM and removing oxoG variants, STRAND includes OXOG along with removing strand bias artifacts, and FFPE includes STRAND but also excludes paraffin artifacts). Rows correspond to sensitivity (SENS), positive predictive value (PPV), F1 measure (F1M) and number of variant calls. The *x*-axis denotes the number of randomly selected unmatched normals to perform the variant calling.

At this point we turned our attention away from overall trends towards individual samples demonstrating large number of variants relative to other cases. We recognized that these were overwhelmingly oxoG. Removal of oxoG AVs at this stage normalized the variant count of the outlier samples with no measurable negative impact on other quality parameters (Figure 2, column OXOG). We then observed that there remained at least one sample with very low PPV and considered the role of strand bias (Figure 3, column STRAND). Filtering remaining variants for strand bias dramatically improved the PPV in a single case and improved the overall average PPV modestly with no measurable impact on sensitivity and slight decreases in the overall decrease in the number of called variants per sample. In a final step, we observed a pattern of variants most consistent with paraffin artifact base substitution. Removal of these variants had no impact on sensitivity and modest improvement in PPV and reduction in the total number of variants called (Figure 3, column FFPE).

It should be noted that the order of filtering was empirically developed. Several criteria are highly correlated, such as paraffin artifact, oxoG and strand bias. Filtering performed in alternative orders would change the marginal impact of each step but would arrive at the same final set of variants. Extending this logic, and in consideration of specific tumor examples we observed that across samples individual filtering criteria had widely different impacts on the total number of variants and the concordance of those variants with the gold standard set.

Selected samples are highlighted in instances where a specific AV had a disproportionate and profound impact (Figure 3). These examples document that the factors associated with quality variant filtering are not uniform across clinical samples. Sample S1 is highlighted for its initial low PPV of 20% in column BASE and UMNQC but once H2M and tumor VAF segmentation filters were applied, PPV reached 70% with 10 UMNs while maintaining 100% sensitivity. Samples S2 and S3, with approximate tumor purities of 90% suffer from low sensitivity once H2MGERM filters were applied. Gold-standard somatic variants were excluded because their allele frequencies were inferred as similar to the local BAF. Sample S4, a hematologic sample had no gold standard variants after applying our filtering criteria while five filtered TO variants remained. While sample S5 maintained 100% sensitivity, after applying filters BASE through FFPE, the PPV plateaued at about 25% because only one gold standard variant remained with an additional three TO variants. Sample S6, under filters BASE through H2MGERM, had over 500 variant calls present in both the gold standard and TO. After applying oxoG filters, the vast majority of both MTN and TO calls were excluded as AVs due to the genome-wide consistent AF ranging between 1% and 3%. Moreover, with only 29 TO calls, 25 of which are refined MTN gold standard calls, sample S6 had a PPV increase from 50% to 86%. At last, sample S7 maintained 100% sensitivity across filtering criteria. The sample harbored about a dozen private GVs, about a dozen hard-to-map variants, 40 oxoG AVs, and 23 strand bias AVs where the noticeable increase in PPV came from the strand bias filtering.

Looking across individual samples, the unfiltered variant calls have varying proportions of private GVs and varying sources of NGS AVs. These factors, along with tumor purity and tumor type, individually impacted each sample’s metrics. But overall, applying the six levels of filtering criteria achieved 94% SENS on average (median of 100%) and 76% PPV on average (median of 78%). It is important to note that if a higher level of sensitivity is desirable, it is possible to reduce the level of filtering. It is also important to note that to achieve these levels of test performance, we did not require annotation filtering such as Polyphen-2 (33) or a similar approach. We reduced the total numbers of variants to a very manageable number with little loss of sensitivity. Further applications, such as the elimination of low impact mutations would allow even more focused review of driver mutations in clinical and other cohorts.

#### False positive calls

Using the filtering strategies described in Figure 2, we observed a plateauing in PPV approaching 80% with 10 UMNs after applying filtering criteria BASE through FFPE due to false positive variants. We then pursued a descriptive evaluation of the remaining 261 false-positive variants in the cohort of 100 samples (Table 3). In UNMASC filtering, we offer strong prejudice to variants that have been reported recurrently in the COSMIC database even though that Figure 1 and other reports suggest that public databases may contain GVs and AVs. About 31 of 261 (12%) variants were retained as false positive because of inclusion in COSMIC despite failing other criteria such as suspicion of germline status (*n* = 14/261). The most cited concern of sequencing the tumor with no MN is false classification of GVs as somatic. Our data demonstrate that only 73/261 (28%) of false positives fall into this category. Importantly, and not unexpectedly a disproportionate fraction of false positive calls overall were insertion-deletion variants (107/261, 41%) suggesting that the user might have a heightened suspicion of indels in the final variant set. In terms of average false positives across samples, 68 samples had 0−3, 27 samples had 4−7 and 5 samples had 8−11. Chromosome X variants presented in 2 male and 3 female samples. UNMASC is designed to retain calls presenting in COSMIC with at least 10 counts regardless of other criteria. However a subset of these calls also presented as various AVs by UNMASC or were germline based on their MN VAF. Among the 14 underlying GVs reported in COSMIC (≥25), ten of them could have been characterized as VIGE using UNMASC’s annotation if COSMIC’s annotation was ignored.

**Table 3.** Summary of false positive variants by reason and variant type. Numbers in the columns are the numbers of false positive variants. Shown in parenthesis are the numbers of patients contributing to the false positive variant number in any class. For example, In row 1 column 2 there were 5 multi-base deletions. Those 5 deletions occurred in 4 subjects, such that 3 subjects had 1 multi-base deletion and 1 subject had 2 multi-base deletions. The total for the row was 8 variants occurring in 6 patients. False-positive variants are divided into base substitutions (Base Sub.), one and multi-base deletions (One-base Del, Multi-base Del), and one and multi-base insertions (One-base Ins, Multi-base Ins). Rows labeled ‘COSMIC, ...’ indicate variants reported in COSMIC at least 10 times and the reason they are false-positive. H2M genes correspond to variants located along genes with identified H2M regions. Non-conforming control AF refers to variants’ matched nVAF appearing non-Mendelian and undetected by H2M. ‘Novel, ...’ rows refer to variants determined to not be GV or AV (oxoG, FFPE, strand bias, H2M), with no variant present in the MN

Reason	BS	DEL (MB, 1B)	INS (MB, 1B)	Total
COSMIC, FFPE		5(4), 2(2)	0(0), 1(1)	8(6)
COSMIC, germline	13(12)		0(0), 1(1)	14(13)
COSMIC, oxoG	2(2)			2(2)
COSMIC, SB	5(5)	2(1), 0(0)		7(6)
Germline	61(38)	6(6), 6(6)		73(44)
H2M Genes	6(5)	2(2), 0(0)	0(0), 1(1)	9(8)
Non-conforming	46(30)	3(3), 2(2)	12(8), 6(6)	69(39)
control AF				
Novel, chr1-chr22	3(3)	2(2), 2(2)	1(1), 0(0)	8(7)
Novel, chrX	5(3)	0(0), 1(1)		6(4)
Novel, in COSMIC		0(0), 3(3)	0(0), 2(2)	5(5)
Other	13(9)	12(11), 21(14)	8(4), 6(6)	60(29)

SB = strand bias, BS = base substitution. MB = multi-base. 1B = one base

Among the 73 GVs, all but four have non-missing VIGE values. Thus the remaining GVs resulted in VIGE probabilities <0.5. With these variants occurring across 44 samples, this suggests a low GV false positive rate of less than two private GVs per sample. Regarding the 69 non-conforming control AF variants, they were characterized by matched control nVAFs ranging between 0.01 and 0.39 and were never called within tumor variant calls when using the MN. These variants share a non-Mendelian characteristic in their nVAF but in a subject-specific manner and thus were not detected as H2M regions. Using the MN as a control would have easily flagged these 69 variants as harboring non-reference bases. The putative false positive novel variants (Table 3) were composed of calls with normal VAF <0.01, VIGE probability <0.5, not identified as H2M (for chr1-chr22 variants) or AV. Mismatch variants considered ‘Other’ had tumor VAFs ranging between 0.015 and 0.123 and appeared to occur almost uniformly across autosomes suggesting possible unaccounted source of artifact.

#### The role of tumor purity

We looked exclusively at the retained variants that underwent all six filtering criteria and after using at least 15 UMNs. Recalling that as tumor purity approaches one, a somatic founder variant (variant found on all copies of a haplotype) and a heterozygous GV becomes indistinguishable. This led us to explore the relationship between tumor purity, derived from the pathologist, and sensitivity as well as number of remaining variants and PPV (Supplementary Figure S11). Of the benchmark samples, two samples, one lymphoma and one hematologic, had missing purity estimates. The remaining samples had purities ranging from 20% to 90%. The figure supported the expected notion that purer samples would result in drops in sensitivity because mutations with VAF close to germline BAFs generally suggests variants to be VIGE rather than somatic, just as in most algorithms. From the second plot, we see the PPV converging toward 85% and with less variability as the number of variants increase. The degree to which variants were incorrectly classified is captured in Supplementary Figure S12, which summarizes the number of false negative variants per sample as well as plots the frequency of tumor purity of samples. Seventy false negative variants across 30 benchmark samples contributed to the drop in sensitivity. Thus across the 100 samples, approximately one variant per sample could be incorrectly classified as germline when in fact it was somatic. These false negative variants composed of 58 base changes and 12 indels spanning 20 high, 42 moderate and 8 low or lesser worst case impacts where 16 of them originated from a single sample with a pathologist reported purity of 90%.

In addition to MTN variant calls, we analyzed the subset of clinically confirmed variants. Out of 215 confirmed variants, all were present in the unfiltered variant calls. However, 29 of them would have been excluded/flagged by UNMASC. Of the 29, one appeared to be a strand bias AV, 20 appeared to be FFPE or oxoG AVs, 4 were heterozygous GVs, 1 appeared to be H2M, and 3 were intronic or synonymous in terms of highest impact. Whether confirmed or non-confirmed mutations, an underlying trade-off exists between detection and classification of mutations.

#### Alternative approaches

For the purposes of indexing our effort to prior efforts, we reviewed a number of approaches (Supplementary Table S1). Among the presented methods, we aimed for self-contained workflows with published and open-source software that did not require supplying a pre-labeled variant set handled similar to the tumor samples of interest, which may not be available to the user, thereby excluding methods SGZ, ISOWN, GATKcan, TOBI and Teer et al.’s method. The remaining methods included Hiltemann et al.’s approach, SomVarIUS and LumosVar 2.0. The BASE and UMNQC steps of UNMASC’s filtering criteria align closely with Hiltemann’s general pipeline by utilizing UMNs. Methods SomVarIUS and LumosVar were run with default arguments, targeted variants were retained, and then annotated with SnpEff for variant effect prediction to retain protein altering impact variants. The Hiltemann workflow calls tumor variants against a set of UMNs and reports the intersection as somatic variants. SomVarIUS runs without UMNs and annotates variants with calculated probabilities of being germline and/or artifact. On the other hand, LumosVar utilizes multiple UMNs to annotate positions in terms of coverage, quality, and depth and models the underlying biology in terms of tumor purity, intra-tumor heterogeneity and copy number states.

**Figure 4.**
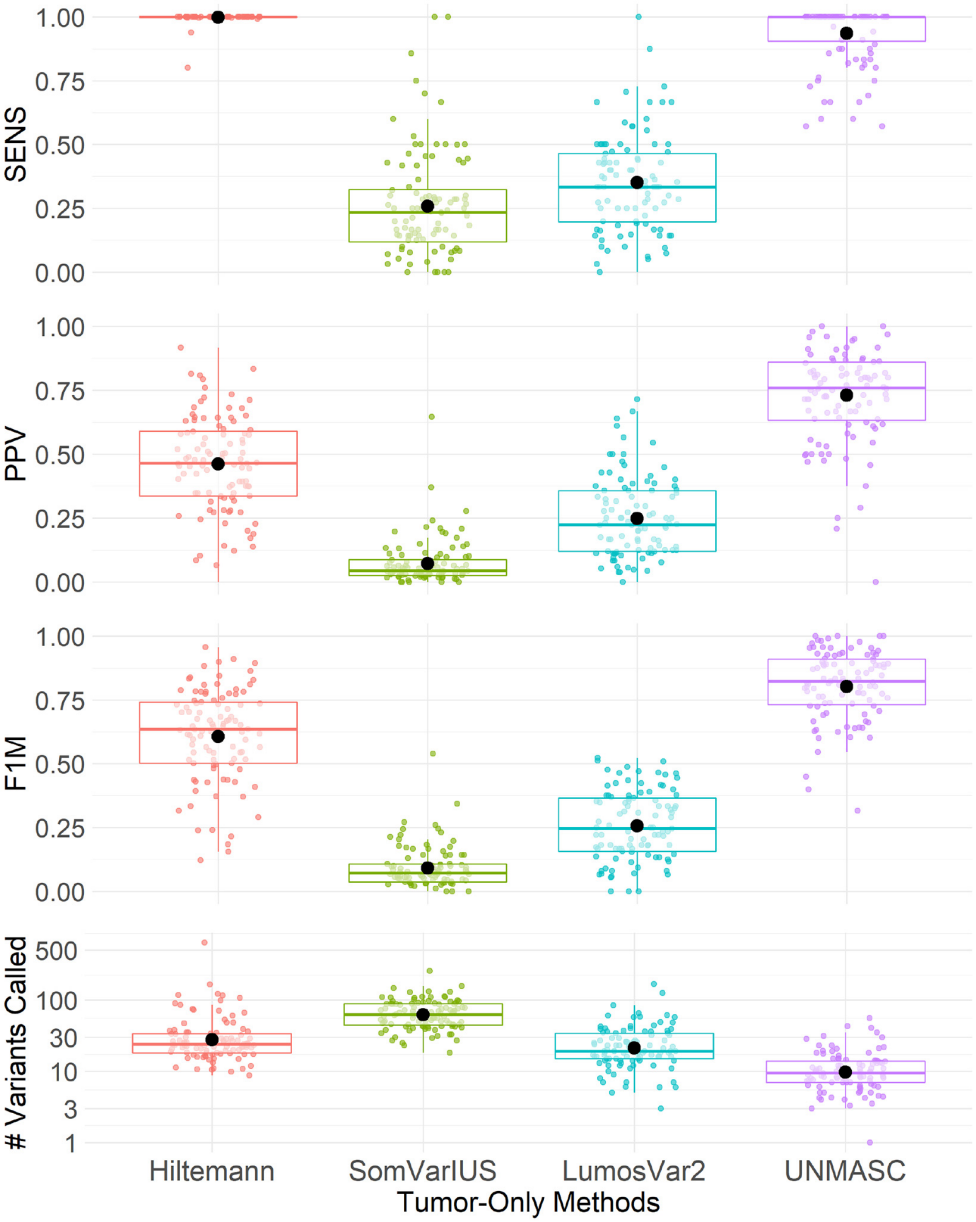
A summary comparison of Hiltemann, SomVarIUS, LumosVar 2.0 and UNMASC performances. Performance metrics include sensitivity (SENS), positive predictive value (PPV), F1-measure (F1M) and number of variant calls after applying filters. The bold lines and points within boxplots correspond to median and mean statistics per method and metric.

We summarized performances in terms of SENS, PPV, F1M and number of variants called (Figure 4). UNMASC and Hiltemann achieve near 100% median sensitivity. Hiltemann attained the second highest mean PPV and F1M metrics. SomVarIUS resulted in an average of 90 variants per case compared to approximately 80 from SomVarIUS, 30 from LumosVar 2.0 and 10 from UNMASC. SomVarIUS’s median sensitivity and PPV were approximately 25% and 7%, respectively, while LumosVar’s median sensitivity and PPV were approximately 35% and 20%, respectively. Hiltemann’s PPV was driven by the presence of unfiltered GVs and AVs. Similarly for SomVarIUS, AVs including strand bias, FFPE, and oxoG composed the majority of false positives. The proportion of GVs was higher in SomVarIUS than in Hiltemann due to misidentified BAF clusters. LumosVar 2.0 made some modest improvements over SomVarIUS, but purity and copy number modeling posed a challenge with the prevalence of low AFs driven by AVs that negatively impact the detection of underlying BAF clusters. Finally, variants called within UNMASC’s identified H2M regions consistently emerged among Hiltemann, SomVarIUS, and LumosVar methods also resulting in lower PPV. Therefore, SomVarIUS and LumosVar 2.0 appear relatively unfavorable for our samples in terms of identifying GVs and AVs.

## DISCUSSION

Despite the importance of variant calling in research and clinical care, as our work has shown, current methodology still lacks precision when applied in an automated fashion. UNMASC provides a new set of tools to address several facets in this field. First, our characterization is systematic with a catalog of expected and unexpected challenges while estimating the relative impact of each on the outcome of accurate variant detection. We demonstrate the empirical challenge that samples have varying numbers of underlying ‘gold-standard’ variant calls and harbor undetected focal copy number alterations, artifacts and private GVs.

A credible set of gold standard variants are required to assess the performance of any methodology in this space. As such we provide an elegant public resource of a large set of tumors with MNs and UMNs. This set contains a wide distribution of sample and tumor types and includes a range of high and lower quality samples representative of clinical cohorts [dbGap phs001713.v1.p1]. Additionally, we offer some of the most conclusive evidence that automated variant calling in tumor with UMNs can be highly sensitive (approaching 100%), highly specific and with very favorable PPV overall. Our approach of scoring variants by various penalties allows the user to consider specific situations where excluded variants might warrant additional review. For example, in a sample suspected of being highly pure for tumor in which somatic variants and contaminating private GVs are difficult to distinguish, the user might decide to retain VIGE variants which are otherwise generally excluded.

While we integrate with public databases which are vital to interpreting variants whether from MNs or UMNs cases, we also provide evidence as to the shortcomings of such database. Variants present in public databases such as COSMIC or dbSNP that have unfavorable penalties by the H2M criteria can be considered with added scrutiny, again either in paired MTN or unmatched cases. This tool does not require elimination of large regions of the genome, entire genes or cataloging of variants. UNMASC’s H2M pipeline can be calculated on the fly and is tuned to the specific sequencing platform and parameters as long as UMNs from the same protocol are available. Although not the primary focus of this manuscript, we suspect that all samples, tumor and normal, can equally suffer in quality from low coverage regions, misaligned reads, and AVs introduced from sample processing. Our work suggests that many normal samples can serve as multiple controls against a tumor and that the nature of variant calling can be dynamically annotated by pooling normal samples. We also demonstrate that each sample may present a unique set of challenges in the number and nature of variants as well as the protocol specific AVs.

### H2M regions and other artifacts

One of the frustrations of reliable variant detection is that many regions of the genome are more challenging than others for mutation calling, yet efficient identification of those regions is elusive. Some users have developed catalogs of such challenging positions whereas a limited number of techniques has proposed computational identification including BlackOps (7) and Umap (8). In the current approach, we develop an efficient method which is platform-specific for high resolution scoring of regions for the unfavorable properties of being H2M, emphasizing that many such regions occur in small neighborhoods that can be identified stochastically by clustering their nVAF. The success of this genome regional clustering approach was extended to define the VIGE variants which share similar properties in a regional manner. The VIGE variants cluster in regions of shared copy number changes in a manner first identified through the B allele frequency techniques of copy number analysis (4,6,22), and we integrate the presence of H2M into this for the first time to our knowledge in the service of variant filtering. Having identified variants with properties of H2M and VIGE allowed us to focus on other variants whose allele frequencies and composition documented useful properties, usually pointing to NGS AVs such as oxoG or paraffin sequencing artifact. Remaining variants, in most cases, are those that were our true positives. The UNMASC autopsy of remaining false positives and negatives documented the need for added concern for indels in the setting of UMN variant detection, as well as raising caution for cases where annotated databases point the user astray. COSMIC, for example contains many false positive variants in H2M locations. Additionally, we dismiss in most cases the concern that private GVs are a major source of false positive when the concept of VIGE is incorporated in variant filtering.

### Limitations

TO variant calling suffers from several limitations. First, the VAF is assumed to follow an underlying distribution that allows us to model genotypes, germline/somatic status or subclonality. For this to work, however, the algorithms require the underlying signal across variants to largely outweigh the noise. This is not always true, especially among indels and in the case of very pure tumors where separation of somatic variants from germline allele frequencies is challenging. As demonstrated by H2M regions, the AF is also a function of the underlying mappability to genomic intervals from a reference genome, whether it is near regions of low complexity or highly polymorphic.

In normal and tumor read count modeling, we chose the binomial over beta-binomial distribution when paired with a discrete uniform (noise) distribution. Through the noise and binomial mixture, highly variable VAFs would be considered noise whereas using a noise and beta-binomial mixture risks classifying them as over-dispersed beta-binomial variants depending on the distribution of noisy variants. In terms of variant classification performance, we aim to maintain high SENS for somatic variants while making small sacrifices to PPV for AVs and GVs.

Identifying segments of copy number aberration using tumor read counts alone relies on a sufficient number of sample-specific germline heterozygous variants. While UNMASC does not rely on detecting changes in total copy number from log R ratios (LRR), derived from matched or UMNs for LRR/BAF joint segmentation, segments can be misidentified due to a lack of GVs. In these regions, UNMASC may misclassify variants, not provide VIGE information, or characterize a segment as noisy (Supplementary Materials Section S4.6). This issue can be overcome when trading read depth for coverage going from targeted capture panel sequencing to whole exome or whole genome sequencing.

A common issue that emerged from previous works is the fact that the ‘denominator’, or number of true-positive variants within a sample impacts the benchmark metric interpretations. A larger number of true-positive variants will provide less spread in PPV and potentially reveals the convergence of PPV. The right plot in Supplementary Figure S11 captures the relationship between PPV and the number of filtered MTN variants which appears to converge toward 80% or more given the scope of the UNCseq™’s targeted capture region.

### Future directions

While the UNMASC benchmark was developed for targeted capture human samples aligned to hg19, we may explore alternate capture versions (whole genome/exome), species and bioinformatic workflows (reference genomes, alignments and variant callers) to assess UNMASC’s protocol-specific features. UNMASC may integrate TO derived copy number calling methods such as SynthEx (34) to refine tumor read count segmentation and improve variant classification, especially in noisy genomic regions where identifying local germline allele frequency remains a challenge. From the benchmark, UMN screening is key to maintaining high sensitivity and further work can be done to identify low quality tumor and normal samples. Using UNMASC, another aspect for future study may look to see if H2M regions are associated with abnormalities from other NGS-based platforms and analyses. Additional biologic constructs could also be incorporated into the work to augment the variant distributions. For example, we could consider the tri-nucleotide context for variant annotation to augment signals associated with linear DNA structure such as certain oxidation artifacts or mutations signals that occur in the context of adjoining bases (35).

The sample set constructed in the current report was intended to include a diverse set of samples with the hypothesis that important differences might be observed as a function of tumor type, gender or other clinical feature. Such patterns were not systematically detected in the current report, although rare outlier samples were observed due to unknown factors. Future cohort design might explore the etiology of such rare outliers for the sources of bias or artifact in the detection of variants such as experimental platform (i.e. sequencing machine or chemistry), sample preparation or other experimental conditions.

## Supplementary Material

zcab040_Supplemental_File
