# Supplementary material for: UNMASC: tumor-only variant calling with unmatched normal controls
Source: NAR Cancer. 2021 Oct 6;3(4):zcab040. doi: 10.1093/narcan/zcab040 (PMC8494212; doi:10.1093/narcan/zcab040)
Supplement: zcab040_Supplemental_File [file zcab040_supplemental_file.pdf]

Supplementary Materials for: **UNMASC: tumor-only variant calling with unmatched normal controls**

Paul Little[1,3], Heejoon Jo[2], Alan Hoyle[3], Angela Mazul[5], Xiaobei Zhao[2,3], Ashley H. Salazar[3], Douglas Farquhar[3,6], Siddharth Sheth[3,7], Maheer Masood[8], Michele C. Hayward[3], Joel S. Parker[3,4], Katherine A. Hoadley[3,4], Jose Zevallos[5], and D. Neil Hayes[2,3,9]

[1]Public Health Sciences, Fred Hutchinson Cancer Research Center
[2]Center for Cancer Research, University of Tennessee Health Science Center
[3]Lineberger Comprehensive Cancer Center, University of North Carolina at Chapel Hill
[4]Genetics, University of North Carolina at Chapel Hill School of Medicine
[5]Otolaryngology Head and Neck Surgery, Washington University School of Medicine
[6]Otolaryngology, University of North Carolina at Chapel Hill School of Medicine
[7]Oncology, University of North Carolina at Chapel Hill School of Medicine
[8]Otolaryngology, University of Kansas Medical Center
[9]Internal Medicine, Division of Hematology-Oncology, University of Tennessee Health Science Center

October 2, 2021

Table of Contents

1 Comparing tumor-only methods features **3**

2 Additional chromosomes with annotations **4**

3 Identifying low quality unmatched normal controls **9**

4 UNMASC **10**

4.1 Notation and Framework . . . 10
4.2 Clustering normal read counts . . . 10
4.3 Normal VAF Segmentation (Hard to map regions) . . . 11
4.4 Strand bias . . . 12
4.5 Identifying oxoG and Paraffin artifacts . . . 12
4.6 tVAF segmentation and inferring germline status . . . 13
4.7 Annotation-based Filters . . . 14
4.8 Workflow . . . 14
4.9 Filtering criteria . . . 16
4.10 Hard to map regions analysis . . . 16

5 Aggregated filtering and one sample classification performance **18**

5.1 Relating SENS and PPV to purity and mutation load . . . 20
5.2 False Negatives . . . 20

1 Comparing tumor-only methods features

Existing methods that address tumor-only or control-free tumor variant calling incorporate some filtering such as on sequencing depth, alternate read depth, variant quality score, read quality, etc. Beyond filtering, several methods can be considered supervised or unsupervised learning approaches. Supervised methods rely on a high quality, pre-labeled, large sample size (subjects and variant calls) dataset of variant calls labeled only as germline or somatic. Unsupervised methods involve clustering variants by modeling the relationship between a variant and known clusters of germline or artifact variant allele frequencies or modeling the underlying biology of the sample (tumor purity, copy number, intratumor heterogeneity, multiplicity). Table S1 provides a breakdown of methods and their features and shortcomings.

Method/Citation	Type	tSEG	ART	UMNs	H2M	RFGS
Hiltemann [1]	FW			✓		
SomVarIUS [2]	FW,UL	✓	(*)			
SGZ [3]	FW,UL	✓				
ISOWN [4]	FW,SL					
LumosVar [5], LumosVar 2.0 [6]	FW,UL	✓	(*)	✓		
GATKcan [7]	FW,SL					
TOBI [8]	FW,SL					
Teer et al. [9]	FW			✓		
UNMASC	FW,UL	✓	✓	✓	✓	✓

Table S1 Representative methods A summary of existing tumor-only methods and their features. Method types include Filtering Workflow (FW), supervised learning (SL), or unsupervised learning (UL). Method features include segmenting tumor copy number (tSEG), characterizing artifacts (ART), using unmatched normals (UMNs), identifying hard to map genomic regions (H2M), re-filtering tumor matched normal gold standard variant calls (RFGS). ART(*) indicates the method incorporated strand bias filtering.

2 Additional chromosomes with annotations

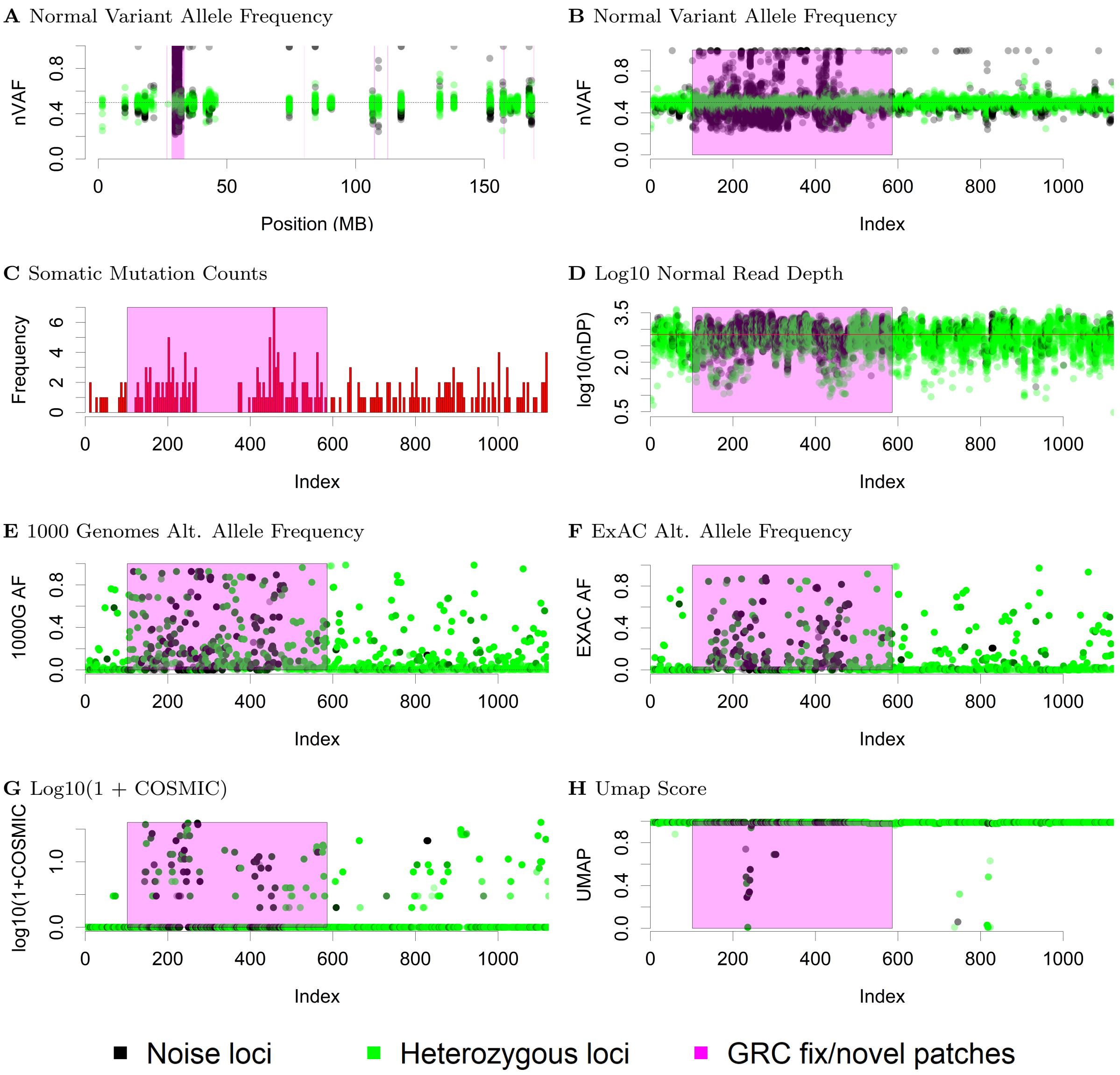

Figure S1 Plots of chromosome 6 with corresponding normal VAFs alongside genome track annotations, total read depth, and frequency of somatic variant calls.

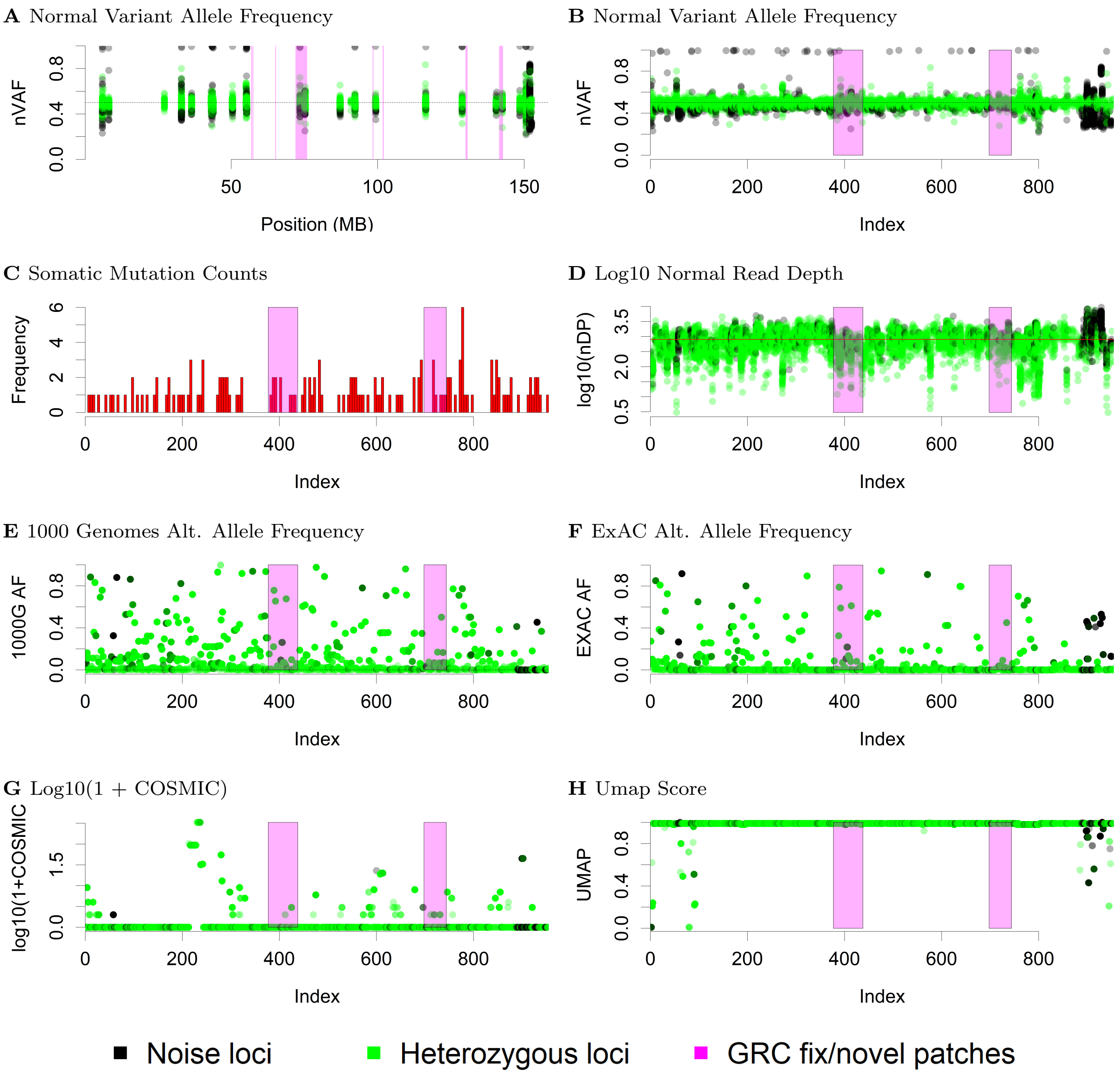

Figure S2 Plots of chromosome 7 with corresponding normal VAFs alongside genome track annotations, total read depth, and frequency of somatic variant calls.

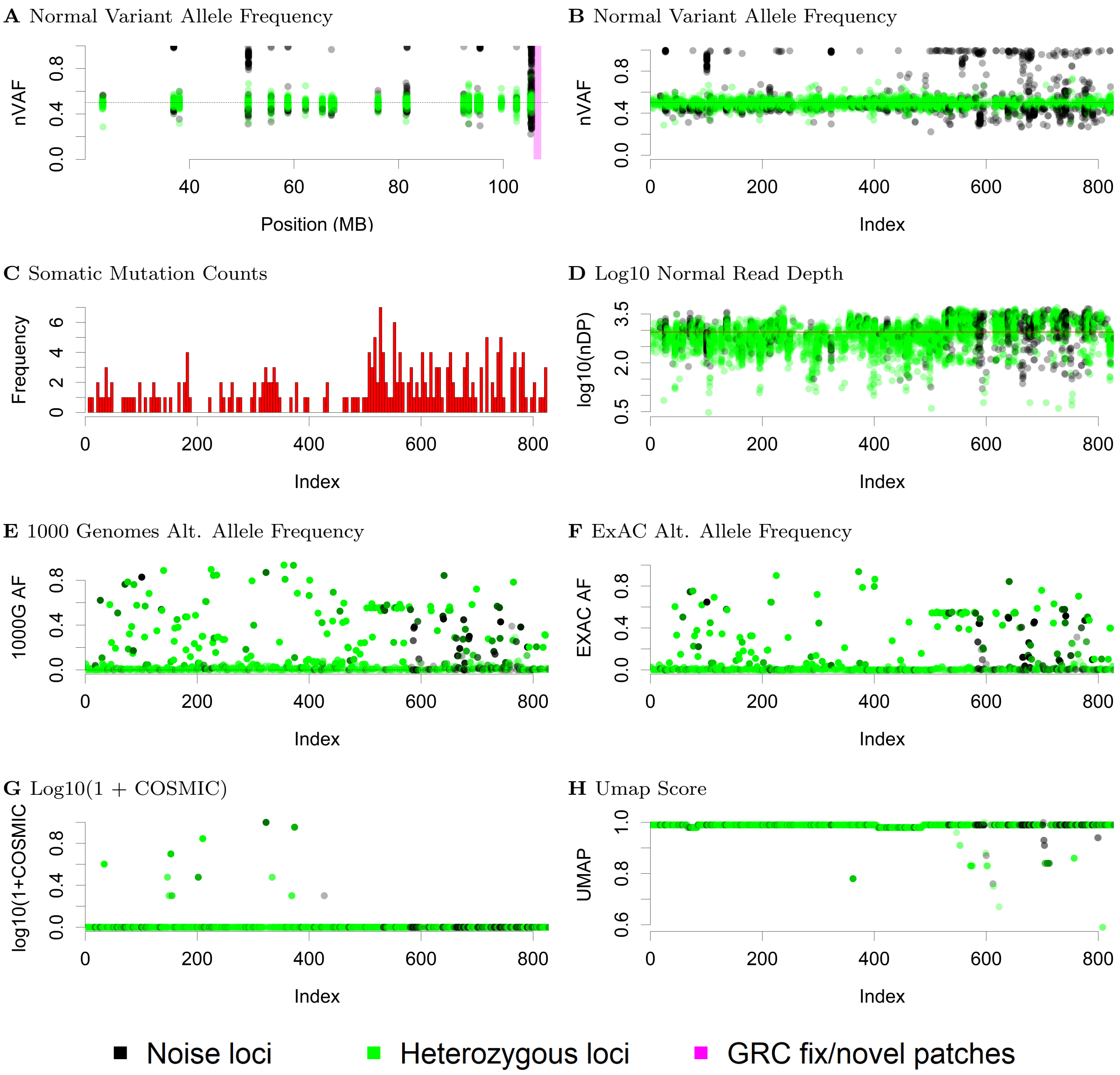

Figure S3 Plots of chromosome 14 with corresponding normal VAFs alongside genome track annotations, total read depth, and frequency of somatic variant calls.

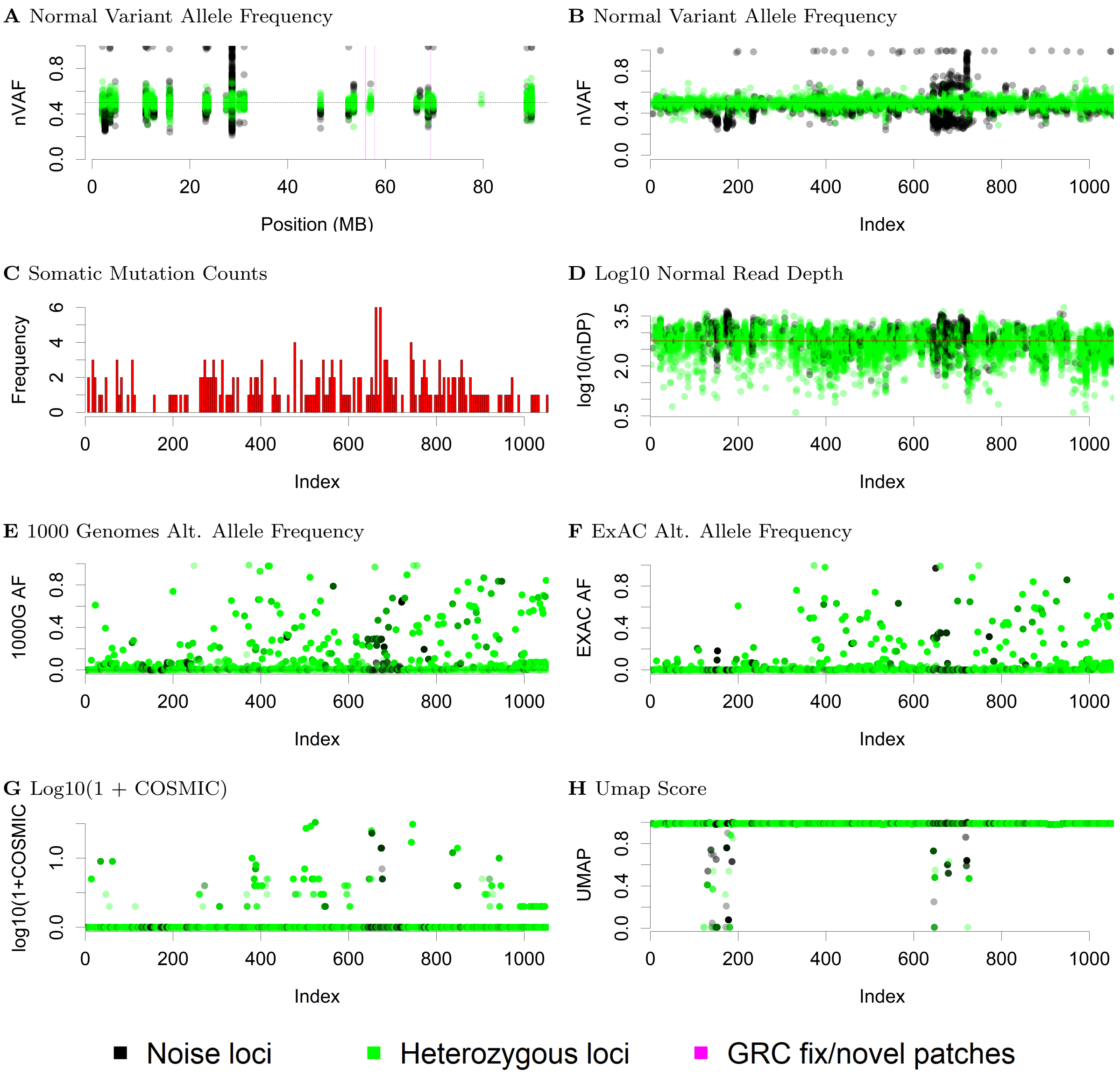

Figure S4 Plots of chromosome 16 with corresponding normal VAFs alongside genome track annotations, total read depth, and frequency of somatic variant calls.

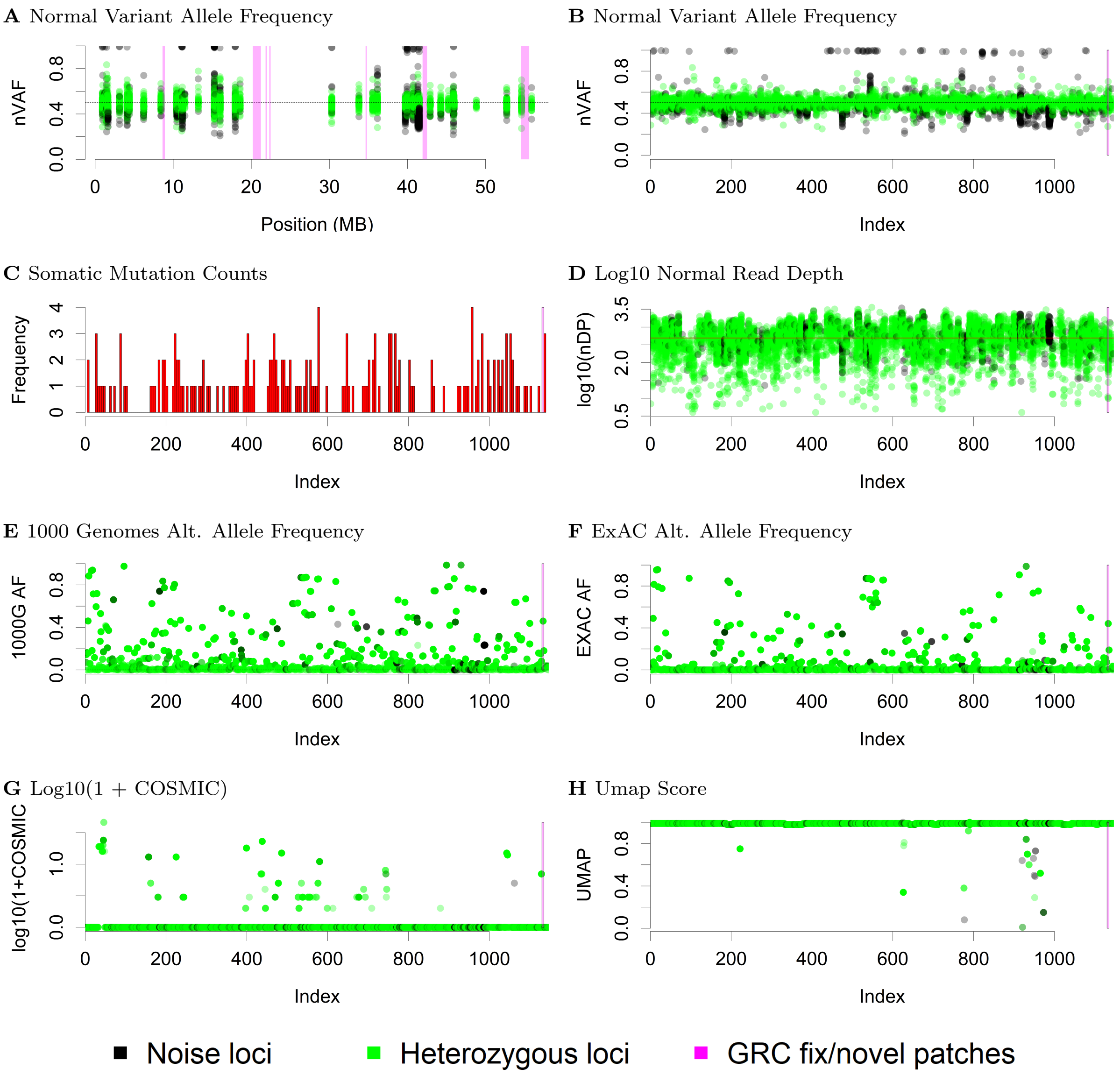

Figure S5 Plots of chromosome 19 with corresponding normal VAFs alongside genome track annotations, total read depth, and frequency of somatic variant calls.

3 Identifying low quality unmatched normal controls

A crucial challenge of tumor-only variant calling with UMNs is to isolate a reliable, high quality set of controls. Otherwise, a low quality sequenced normal control could consistently miscall a variant leading to a decrease in sensitivity. From among over 1,000 tumors with matched normal samples, we plotted and clustered their Picard hsMetrics to identify low quality normals to exclude from the UMN set. While this manuscript does not focus on our approach to identify outlier samples and retain high quality UMNs, this aspect can be further explored.

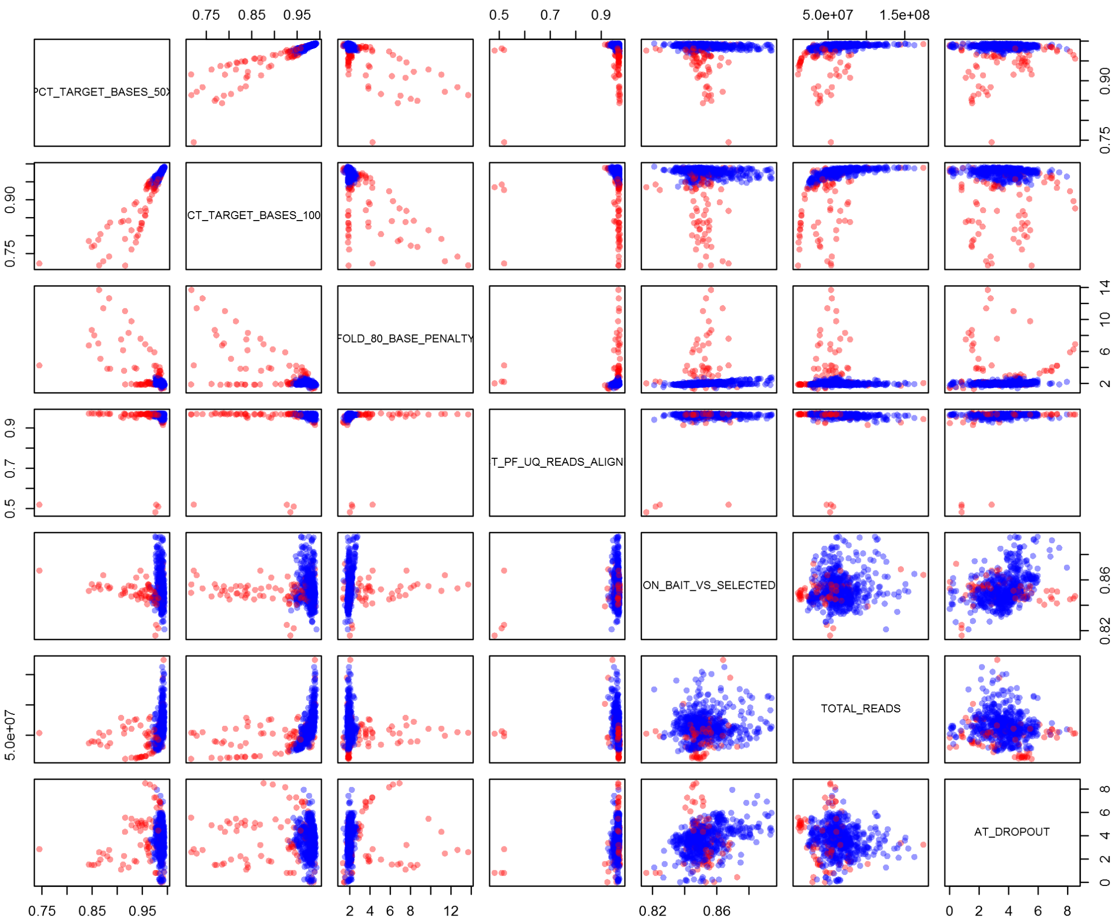

Figure S6 UMN quality metrics. Picard hsMetrics colored by inferred cluster (blue for non-outlier and red for outlier) to identify and exclude lower quality normal v8 samples. PCT_TARGET_BASES_50X and PCT_TARGET_BASES_100X denote the percent of target bases covered to 50X and 100X coverage or more, respectively. FOLD_80_BASE_PENALTY denotes the fold over-coverage necessary to increase 80% of bases in non-zero coverage targets to the mean coverage level in those targets. PCT_PF_UQ_READS_ALIGNED denotes the fraction of passing filter reads that are unique and align to the reference genome. ON_BAIT_VS_SELECTED denotes the fraction of bases on or near baits that are covered by baits. TOTAL_READS denotes the total number of reads assigned to the index. AT_DROPOUT denotes a measure of how regions with less than 50% GC content are undercovered relative to mean coverage.

4 UNMASC

4.1 Notation and Framework

For each tumor and UMN, a set of variant calls are generated. Each variant call is characterized by a pair of normal and tumor read counts. We describe the procedure applied to the UMN read counts. In a given genomic position, a variant present in the tumor that is not present in the MN would be considered somatic. And if a variant allele was considered present in the MN, the position in the tumor would be ignored. However, without the MN, the status of variant alleles detected at a position is unknown when calling somatic variants in the tumor sample. Using a set of UMNs, our procedure aims to characterize the nature of variant calling at a position or within a genomic interval.

All of the proposed methods described below to generate variant call features rely on modeling read counts with a mixture model composed of a discrete uniform distribution and binomial densities with success probability 0.5, p, and $1-p$ for $0 < p < 0.5$. Let b denote the bth genomic locus, where $b = 1, \dots, B$. Let (A_b, R_b) denote the bth locus's normal alternate and reference read counts, respectively. Letting $T_b = A_b + R_b$ denote the total depth, the VAF is A_b/T_b. Let C_b denote the unobserved classification of the bth locus. Let $\mathcal{C}$ denote the set of possible classifications. Let $\boldsymbol{\theta} = (\boldsymbol{\pi}_{\mathcal{C}}, p)$ denote the set of underlying parameters where $\boldsymbol{\pi}_{\mathcal{C}}$ denotes the vector of mixture proportions and $\sum_{c \in \mathcal{C}} \pi_c = 1$. Let $Bin(n,p)$ denote a binomial distribution with n trials and success probability p. In addition, let $1\{\cdot\}$ denote the indicator function where $1\{A\}$ equals 1 if A and zero otherwise. The mixture distribution is characterized as

$$\begin{aligned} P(A_b|T_b;\boldsymbol{\theta}) &= \sum_{c \in \mathcal{C}} P(C_b = c, A_b|T_b;\boldsymbol{\theta}) \\ &= \sum_{c \in \mathcal{C}} P(C_b = c|T_b;\boldsymbol{\theta}) P(A_b|T_b, C_b = c;\boldsymbol{\theta}) \\ &= \sum_{c \in \mathcal{C}} \pi_c P(A_b|T_b, C_b = c;\boldsymbol{\theta}) \end{aligned}$$

where

$$P(A_b|T_b, C_b = c;\boldsymbol{\theta}) = \begin{cases} 1/T_b & \text{if } c = 1 \\ Bin(T_b, 0.5) & \text{if } c = 2 \\ Bin(T_b, p) & \text{if } c = 3 \\ Bin(T_b, 1-p) & \text{if } c = 4 \end{cases}.$$

The marginal likelihood is $\prod_{b=1}^{B} P(A_b|T_b;\boldsymbol{\theta})$ which is optimized by introducing latent variables $C_1, \dots, C_B$ and optimizing the expected complete log-likelihood through the EM algorithm.

4.2 Clustering normal read counts

In normal samples, we assume each locus is drawn from a diploid genome and thus there are three underlying genotypes (AA, AB, BB) or probability densities to consider. Positions with genotype AA, AB, and BB tend to have a VAF near zero, VAF near 0.5, and VAF near one, respectively. To capture positions that do not reflect the three possible genotypes, we include a "noise" density. This density encapsulates sequencing and alignment phenomenon that disagree with the underlying biology. Treating these four underlying classes as unknown, we can cluster their normal read counts. We model the B pairs of read counts with a mixture distribution consisting of six proposed densities. The first density accounts for noise genotypes, the second density accounts for heterozygous genotypes, the third density accounts for homozygous reference genotypes with expected VAF p, and finally, the fourth density accounts for homozygous alternate genotype with expected VAF $1-p$.

Using the notation above, let $\mathcal{C} = \{1, 2, 3, 4\}$. Let $\boldsymbol{\theta} = (\boldsymbol{\pi}_{\mathcal{C}}, p)$. After the EM algorithm converges, we retain all loci classified to classes $c = 1, 2$, in other words, classified as noise or heterozygous, respectively, for subsequent segmentation. Assuming there are Z UMNs, this procedure is repeated across each set of the tumor variant calls' UMN read counts.

4.3 Normal VAF Segmentation (Hard to map regions)

Taking the union of positions across all Z variant calls and retained positions labeled noise or heterozygous, we perform segmentation. The goal is to construct a sparse segmentation, by taking advantage of the ordered loci, where each genomic interval is characterized by the proportion of loci with VAF deviating from 0.5. Suppose there are B_Z unique and ordered genomic positions remaining where $b = 1, \ldots, B_Z$ indexes the relative position and each position has at most n_b pairs of normal read counts indexed by UMN z. Let $V_{bz} = 1$ and $V_{bz} = 0$ denote the indicator of a variant called and classified at noise or heterozygous or not called, respectively, at locus b for the zth UMN. For example, position b was called a variant among all Z UMNs but only UMNs 1, 2, and 5 have corresponding normal read counts classified as noise or heterozygous, and so $n_b = \sum_{z=1}^{Z} V_{bz} = 3$.

As a data-driven annotation, our proposed segmentation scheme is designed to identify genomic intervals where the normal VAF does not match our expectations, which we refer to as hard-to-map (H2M) intervals. Generated through simulation, Figure S7 illustrates the goal of this segmentation. Figure 1b illustrates this phenomenon observed along chromosome 1.

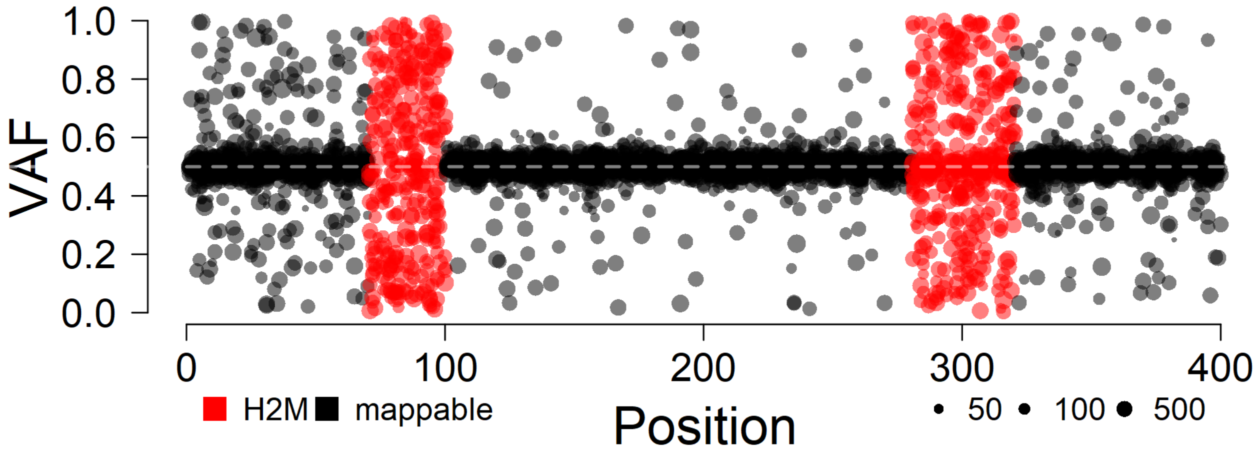

Figure S7 Simulated variant allele frequencies (VAF) with segments colored by mappable ($\pi < 0.5$) and hard to map (H2M) ($\pi \geq 0.5$) regions and point sizes correspond to total depth. From left to right, the five segments have noise parameter values of $\pi_1 = 0.2$, $\pi_2 = 0.95$, $\pi_3 = 0.05$, $\pi_4 = 0.7$, and $\pi_5 = 0.1$.

Circular binary segmentation (CBS) [10] was not utilized because the algorithm is optimized for piecewise constant segments and pre-smoothed univariate data. CBS would not account for sequencing depth and not allow us to incorporate segments with expected VAFs centered around 0.5 and identify noisier genomic segments. The aim of our approach is to quantify the proportion of noisy loci per segment.

Using Figure 1b as motivation for our segmentation method, suppose segment i is composed of normal read counts with indicies b bounded by start and end indices s_{i-1} and s_i, respectively. Using the notation defined, the segment-specific parameter is $\boldsymbol{\theta}_i = (\pi_i)$ and $\mathcal{C} = \{1, 2\}$ where

$$P\left(A_{bz}|T_{bz};\pi_i\right) = \pi_i \frac{1}{T_{bz}} + (1-\pi_i) Bin(T_{bz}, 0.5).$$

Within segment i, let

$$l_i = \log\left[\prod_{b=1+s_{i-1}}^{s_i} \prod_{z=1}^{Z} \left\{P\left(A_{bz}|T_{bz};\pi_i\right)\right\}^{V_{bz}}\right],$$

$m_i = 1\{\pi_i > 0\} + 1\{1-\pi_i > 0\}, N_i = \sum_{b=1+s_{i-1}}^{s_i} \sum_{z, V_{bz}=1} V_{bz}$ denote the observed likelihood, model size, and number of pairs of normal read counts, respectively. Due to possible local optimum, the goal within each segment is to retain the clustering result with largest BIC. Given I segments with fixed segmentation bounds $s_0, s_1, \ldots, s_I$,

where $s_0 = 0$ and $s_I = B_Z$, the objective function to be maximized is

$$\mathcal{L}(I) = 2\sum_{i \in I} l_i - \log\left(\sum_{i \in I} N_i\right)\sum_{i \in I} m_i$$

Notice that calculating $\mathcal{L}(I)$ requires EM algorithms run per segment. Let $I_{(t)}$, $I_{(t)}^{+}$, $I_{(t)}^{-}$, $I_{(t)}^{\sim}$ denote the current segmentation, current segmentation with added segment, current segmentation with dropped segment, current segmentation with adjusted segment, respectively. Here is our segmentation algorithm.

Algorithm 1 VAF segmentation

```
t ← 0, initialize segmentation I_(0) and calculate L(I_(0)).
while true do
    At iteration t, propose adding to, dropping from, and adjusting the current segmentation.
    if L(I+_(t)) > L(I_(t)) then
        L(I_(t+1)) = L(I+_(t))
    else if L(I-_(t)) > L(I_(t)) then
        L(I_(t+1)) = L(I-_(t))
    else if L(I~_(t)) > L(I_(t)) then
        L(I_(t+1)) = L(I~_(t))
    else
        Exit the segmentation
    end if
end while
```

Once the segmentation of the normal read counts terminates, the relative positions b are mapped back to genomic positions to annotate genomic intervals by their locally estimated noise parameter $\widehat{\pi}_i$. Since $\widehat{\pi}_i$ represents the estimated proportion of UMN VAFs drawn from the discrete uniform distribution, we consider segments with $\widehat{\pi}_i > 0.5$ as hard-to-map, otherwise they are mappable, to reflect a simple majority of VAFs deviating from 0.5.

4.4 Strand bias

Strand bias detection is conducted by determining if the allele frequencies of the forward and reverse strands are consistent with one another at a given locus. Using Rsamtools [12], forward and reverse strand read counts harboring alternate and reference bases are obtained per tumor variant call. UNMASC implements the Fisher exact test and the outputted annotation includes the strand-specific alternate and reference read counts along with the strand bias p-value.

4.5 Identifying oxoG and Paraffin artifacts

Given existing works [13–15] that have studied specific artifacts, we attempt to incorporate their findings into our workflow. The oxoG artifact has been characterized by a sample's relative abundance of C>A (and G>T) base substitutions over all other base substitutions. If these variants occurred at approximately the same time or with the same proportion of reads after library amplification, then we expect them to occur with VAFs centered around a single mean regardless of somatic copy number alterations. We also expect that paraffin induced variants, previously characterized by a sample's relative abundance of C>T and G>A base substitutions and indels should also follow a similar pattern with their own respective allele frequency.

These artifacts are identified by modeling the tumor variant calls made by the zth UMN with a mixture of a discrete uniform density and binomial density for non-artifact and artifact variants, respectively. Using the above

notation, let $\boldsymbol{\theta} = (\pi_1, p)$ and $\mathcal{C} = \{1, 3\}$. The model is characterized as

$$\begin{aligned} P\left(A_b|T_b;\boldsymbol{\theta}\right) &= P\left(C_b = \text{noise}, A_b|T_b;\boldsymbol{\theta}\right) + P\left(C_b = \text{artifact}, A_b|T_b;\boldsymbol{\theta}\right) \\ &= \pi_1 \frac{1}{T_b} + (1-\pi_1)Bin(T_b, p) \end{aligned}$$

where variants clustered to the binomial density are considered artifact (labeled "signal"), otherwise the variants are considered not artifact (labeled "noise"). To identify oxoG artifacts, we cluster variants that meet our filtering criteria (total depth, Qscore, 1000 Genomes, ExAC, targeted, and lacking strand bias) with base substitutions C>A and G>T and tumor VAF less than a maximum expected tumor VAF, hopefully less than 0.5. Similar to oxoG, to identify paraffin artifacts, we cluster variants that meet the above filtering criteria but with indels and base substitutions C>T and G>A. This clustering procedure is repeated over each of the Z UMN tumor variant calls to be aggregated together. Conceptually, this procedure is applied to each UMN's set of tumor variant calls to assess the consistency of the artifact distribution.

In addition to clustering variants with the specific base substitution or indel, we make use of the above clustering results in an attempt to identify additional artifacts based on those that share a similar tumor VAF. Taking variants with the same filtering criteria but not satisfying the ExAC, 1000 Genomes, or base substitution/indel criteria, we infer their status as either artifact (labeled "signal like") or not artifact ("noise") using the maximum posterior probability characterized as

$$P\left(C_b = \text{artifact}, A_b \middle| T_b; \widehat{\boldsymbol{\theta}}\right) = \frac{(1-\widehat{\pi}_1)Bin(T_b, \widehat{p})}{\widehat{\pi}_1(1/T_b) + (1-\widehat{\pi}_1)Bin(T_b, \widehat{p})}.$$

The remaining variants that do not undergo artifact clustering or prediction are labeled "NA".

4.6 tVAF segmentation and inferring germline status

With strand and artifact annotations inferred, the remaining tumor-only variants can be segmented to identify local allele frequency patterns of GVs. Without excluding potential AVs, our segmentation algorithm could be distorted by clusters of artifacts at similar allele frequencies.

Due to loci repeatedly called across the Z UMNs, we take the unique union of tumor variant calls. There are many tumor variants with VAFs near 0 and 1. These variants are removed before filtering. Within segment i, to account for possible copy number alterations, the mixture model is composed of a discrete uniform density, a binomial density centered around 0.5, and a pair of binomial densities centered around p_i and $1-p_i$ where $p_i < 0.5$ to identify somatic mutations, segments of allelic balance, and segments of allelic imbalance, respectively. Our proposed segmentation algorithm does not rely on GDs to identify and segment GVs. Let $\boldsymbol{\theta}_i = (\pi_{i1}, \pi_{i2}, \pi_{i3}, p_i)$. The proposed mixture model for each unique tumor variant read count within segment i is characterized as

$$\begin{aligned} P\left(A_b|T_b;\boldsymbol{\theta}_i\right) &= \sum_{c=1}^{4} P\left(A_b, C_{ib} = c|T_b;\boldsymbol{\theta}_i\right) \\ &= \sum_{c=1}^{4} P\left(C_{ib} = c|T_b;\boldsymbol{\theta}_i\right) P\left(A_b|T_b, C_{ib} = c;\boldsymbol{\theta}_i\right) \\ &= \sum_{c=1}^{4} \pi_c P\left(A_b|T_b, C_{ib} = c;\boldsymbol{\theta}_i\right) \end{aligned}$$

where $\sum_{c=1}^{4} \pi_{ic} = 1$. Let $l_i = \log\left[\prod_{b=1+s_{i-1}}^{s_i} P\left(A_b|T_b;\boldsymbol{\theta}_i\right)\right]$, $m_i = 1\{\pi_{i1} = 1\} + \sum_{c=1}^{4} 1\{\pi_{ic} > 0\} - 1 + 1\{\pi_{i2} > 0\} + 1\{\pi_{i3} + \pi_{i4} > 0\}$, $N_i = s_i - s_{i-1}$ denote the observed likelihood, model size, and number of tumor read counts clustered, respectively, within segment i. Given I segments with fixed segmentation bounds $s_0, s_1, \ldots, s_I$, where $s_0 = 1$ and $s_I = B_Z$, the objective function to be maximized is

$$\mathcal{L}\left(I\right) = 2\sum_{i \in I} l_i - \log\left(\sum_{i \in I} N_i\right) \sum_{i \in I} m_i$$

The steps of tumor VAF segmentation algorithm are identical to the steps of normal VAF segmentation, the only difference being the mixture model proposed for tumor versus normal read counts. The output from this algorithm will contain segment start and end positions, where segment i is characterized by parameters $\widehat{\boldsymbol{\theta}}_i$, to be used for inferring the germline status of candidate somatic variants.

Before characterizing a variant relative to its local segment, we need to determine if the segment itself is in allelic balance or imbalance to determine the B allele frequency. If $\widehat{\pi}_{i1} = 1$, this indicates that all loci within a segment are classified as noise and thus the allele status of that segment is unknown. But if $\widehat{\pi}_{i1} < 1$, the ith segment is considered to be in allelic balance if $\widehat{\pi}_{i2} \geq 0.5$, otherwise the segment is in allelic imbalance. Given the allelic status for a segment, the posterior probability can be calculated of a variant not belonging to the distribution of local GVs. The poster probability of a variant not clustering to the distribution of local GVs or (1 − GPP) is $P\left(\text{not BAF}, A_b \middle| T_b; \widehat{\boldsymbol{\theta}}_i\right)$. This quantity is NA if $\widehat{\pi}_{i1} = 1$, 0 if $\widehat{\pi}_{i1} < 1, \widehat{\pi}_{i2} \geq 0.5, \frac{A_b}{T_b} \geq 0.5$, $\sum_{c=1,3,4} P\left(C_{ib} = c, A_b \middle| T_b; \widehat{\boldsymbol{\theta}}_i\right)$ if $\widehat{\pi}_{i1} < 1, \widehat{\pi}_{i2} \geq 0.5, \frac{A_b}{T_b} < 0.5$, 0 if $\widehat{\pi}_{i1} < 1, \widehat{\pi}_{i2} < 0.5, \frac{A_b}{T_b} \geq \max(\widehat{p}_i, 1 - \widehat{p}_i)$, and $\sum_{c=1,2} P\left(C_{ib} = c, A_b \middle| T_b; \widehat{\boldsymbol{\theta}}_i\right)$ if $\widehat{\pi}_{i1} < 1, \widehat{\pi}_{i2} < 0.5, \frac{A_b}{T_b} < \max(\widehat{p}_i, 1 - \widehat{p}_i)$ where

$$P\left(C_{ib} = c, A_b \middle| T_b; \widehat{\boldsymbol{\theta}}_i\right) = \frac{\widehat{\pi}_{ic} P\left(A_b \middle| T_b, C_{ib} = c; \widehat{\boldsymbol{\theta}}_i\right)}{\sum_{c'=1}^{4} \widehat{\pi}_{ic'} P\left(A_b \middle| T_b, C_{ib} = c'; \widehat{\boldsymbol{\theta}}_i\right)}.$$

For females, chromosome X's tumor VAF was segmented to determine local BAF status relative to candidate variant loci whereas no segmentation along chromosome X was performed for males to characterize the relation between tVAF to BAF.

4.7 Annotation-based Filters

Given a set of UMNs, the aim is to extract the set of (1) exonic/coding, (2) on-target, (3) high quality, and (4) somatic variant candidates. Strand bias, oxoG, and FFPE results are initially annotated for all variants. For a given tumor sample, we begin by pooling together all variant calls across all tumor/normal pairs. For (1), the exonic/coding variants based on SnpEff and GENCODE annotations with the highest impact or worse case scenario are retained. For (2), using the corresponding target capture BED file, on-target variants are retained. For (3), variants with a total tumor read depth of 10 and where the 25th percentile in normal read depth across UMNs is at least 10, harbor at least 5 alternate reads, and 25th percentile in Qscore across UMNs is 30 were retained. If a variant did not meet these thresholds but was reported at least 10 times in COSMIC, not reported in 1000 Genomes and not reported in ExAC or with a population frequency less than 0.005, they would also be retained. Moreover, through clustering of the normal read counts we have inferred homozygous reference (G=0), homozygous alternate (G=2), heterozygous (G=1), or noise (G=NA) status. Among variants in positions classified as G=0 among 50% of UMNs and called among 90% of UMNs. For (4), variants not reported in 1000 Genomes and not in ExAC or with a population frequency less than 0.005 are retained. The final set of variants that met all of the above criteria are referred to as candidate somatic variants. Through tumor VAF segmentation, UNMASC calculates and annotates them with their posterior probability of not sharing the distribution of the local segmented B allele frequency.

4.8 Workflow

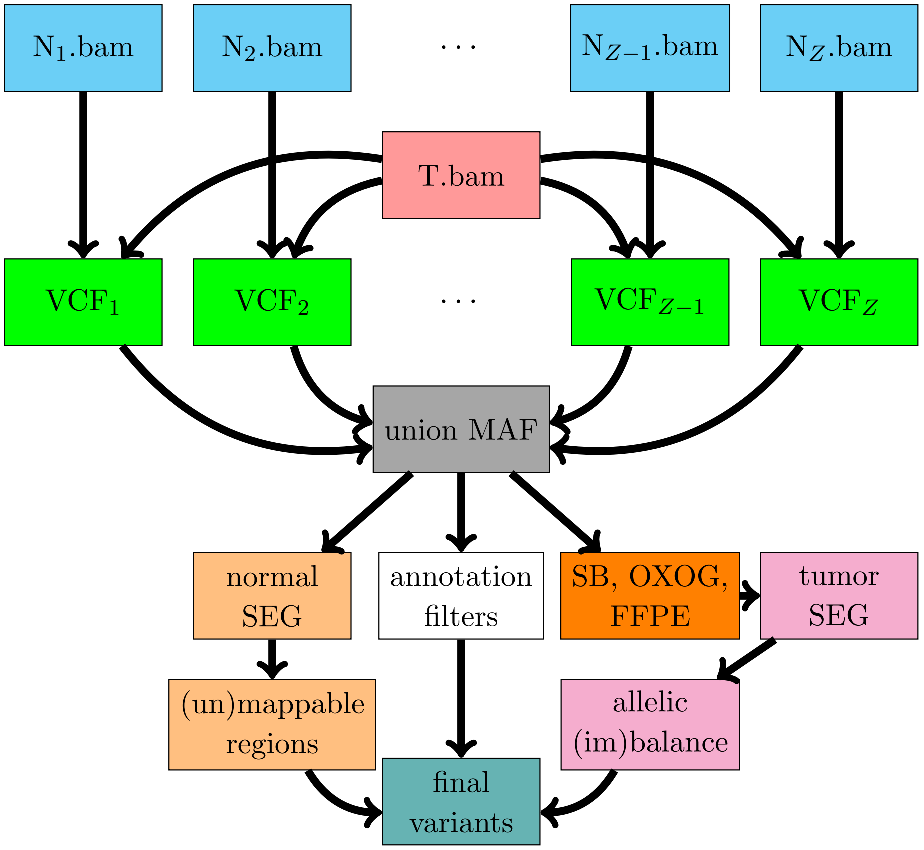

Figure S8 UNMASC workflow for a single tumor sample. The UNMASC workflow starting with Z unmatched normals (UMNs) bam files (N_j.bam) and a tumor sample's bam file (T.bam) as inputs and resulting in annotated variant calls (VCs) for manual inspection and filtering, where $j = 1, \ldots, Z$. The vcf files (VCF_j) are generated by Strelka and annotated with genes, COSMIC counts, ExAC, SnpEff, Variant Prediction, 1000 Genomes by Oncotator. The vcfs are combined (union MAF) and labeled as ON or OFF target, according to a supplied bed file. UMN read counts are clustered to infer noise or genotype status. Combining UMN genotype status, Oncotator annotations, tumor/normal read depth, qscore, and variant call frequency, we obtain a set of candidate somatic variant calls. Using only noise and heterozygous loci, we segment the corresponding read counts to infer mappable and unmappable genomic segments per chromosome. Tumor strand-specific read counts are extracted with samtools for strand bias annotation. In addition, strand bias (SB), OXOG and FFPE analyses are conducted to further infer artifact status before segmentation to avoid misclassifying germline variants. Non-strand bias, non-OXOG, and non-FFPE tumor loci read counts are segmented. Candidate variant calls are further annotated by tumor and UMNs segmentation results.

4.9 Filtering criteria

Table S2 UNMASC filtering criteria applied to our benchmark set.

Criteria	Feature	Definition	Filter/Annotation
BASE	COSMIC	Cosmic count	≥ 10
	nDP_stat	25th percentile UMN read depth	≥ 10
	tDP	Tumor read depth	≥ 10
	tAD	Tumor alternate read depth	≥ 5
	Q_stat	25th percentile UMN Qscore	≥ 30
	EXAC_AF	ExAC allele frequency	≤ 0.005
	1000G_AF	1000 Genomes allele frequency	≤ 0.005
	EXONIC	Isolate non-synonymous, exonic, low/moderate/high impact	snpEff and/or GENCODE annotation
	TARGET	Identify on-target variants	Target capture bed file
UMNQC		Remove lower quality UMNs	
H2MGERM	H2M	Prop. non-conforming allele frequency	< 0.5
	VIGE	Prob. tVAF and local BAF are the same	≤ 0.5
OXOG	OXOG	Frequency of oxoG classification	% "signal" or "signal-like" $\leq$ 50%
STRAND	SB	Strand Bias Fisher test probability	> 0.05
FFPE	FFPE	Frequency of FFPE classification	% "signal" or "signal-like" $\leq$ 50%

4.10 Hard to map regions analysis

Table S3 UNCseq^{TM} targeted genes harboring identified hard to map (H2M) intervals. The count represents the number of tumor samples that identified a portion of the gene as H2M. A gene is included if at least 20 tumor samples identified a H2M interval within the gene body. And thus, even though a gene is listed does not mean the entire gene body is hard to map.

Chr	Genes
chr1	FCGR2B(55);GSTM1(35);NOTCH2(100);PDE4DIP(100)
chr2	ANTXR1(22)
chr3	FANCD2(96);VHL(79)
chr4	TLR3(25)
chr5	CDH6(20);FGFR4(21);RICTOR(69);TERT(45)
chr6	HLA-A(95);HLA-B(100)
chr7	ACTR3B(65);KMT2C(100)
chr8	RUNX1T1(34)
chr9	GNAQ(35);MLLT3(37)
chr10	GATA3(21);NUTM2A(52);PTEN(28)
chr11	HRAS(35);LRRC56(28)
chr12	KDM5A(36);NACA(61)
chr13	FLT1(28);MTUS2(86);RB1(31)
chr14	AHNAK2(99);NKX2-1(39)
chr15	IGF1R(35);NTRK3(50)
chr16	GDPD3(52);IL21R(21);ORC6(60);PDPK1(97);<br>SULT1A1(98);VKORC1(33);ZNF668(24)
chr17	NCOR1(22)
chr18	SMAD4(20);TCF4(70)
chr19	CTC-490E21.12(63);CYP2A6(97);CYP2B6(24)
chr20	ASXL1(20)
chr21	RUNX1(52)
chr22	BCR(98);COMT(31);CYP2D6(82);CYP2D7P1(43);<br>DERL3(47);EP300(28);GSTT1(42);SMARCB1(46)

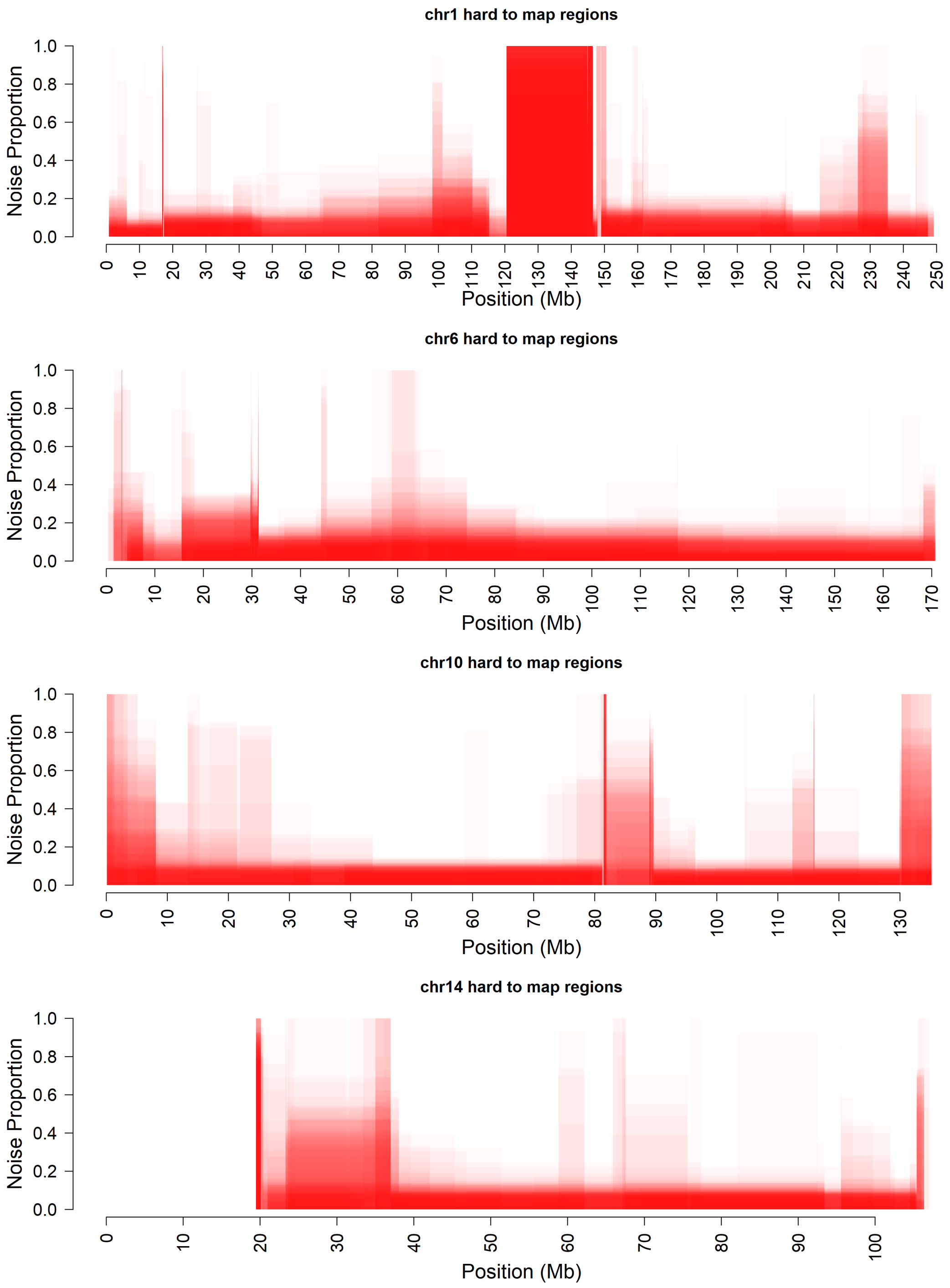

Figure S9 An overlay of 100 benchmark nVAF segmentations along chromosomes 1, 6, 10, and 14. The x-axis denotes the genomic position in megabases and the y-axis denotes the proportion of loci within a segment classified as noise. Darker color intensities correspond to more consistently identified regions detected at similar noise proportions. For example, the centromere region on chr1 is consistently detected with 100% of variants classified as noisy for segmentation. In addition, a narrow region less than 20 megabases on chr1 is also consistently identified as hard to map. Similar cases of wide and narrow H2M regions are displayed.

5 Aggregated filtering and one sample classification performance

We observed the combined effects of applying UNMASC's filtering criteria, varying the number of unmatched normal controls, and varying the threshold for proportion of times a variant was called across UMNs (25%, 50%, 75%, 90%, and 100%). This was conducted by sampling (without replacement) pools of UMNs with sizes ranging from one UMN to all available UMNs. A pool size was sampled ten times to capture any variability in SENS, PPV, and number of filtered variant calls each distinct set of UMNs might result in. The six cumulative layers of applied filtering criterion were applied to both the TO variants and TMN variants.

Criteria BASE corresponds to applying the annotation-based filters including depth, Qscore, on-target, exonic, Cosmic, ExAC, and 1000 Genomes filters. Criteria UMNQC includes Criteria BASE specifications but removes three specific UMNs that resulted in decreases in sensitivity for a subset of samples from Criteria BASE. This was due to drops in Qscore and depth below our pre-defined thresholds and a handful of variants not called by one of the three UMNs. We attributed these undesired properties to outlier metrics in the percent of bases covered and fold 80 base change from Picard hsMetrics. For one sample presented in Figure S10, SENS was restored to 100% while PPV suffered a small decrease.

Criteria H2MGERM builds on Criteria UMNQC and filters variants by integrating the segmentation results. More specifically, variants falling in H2M regions and variants inferred to be germline are excluded. This resulted in a significant gain in PPV, reflected by an overall decrease in filtered variant calls, while maintaining SENS at 100%. Next, Criteria OXOG incorporates Criteria H2MGERM by excluding variants UNMASC deemed to be oxoG artifacts. In Figure S10, the dotted black line represents the corresponding criteria's number of filtered TMN variant calls (fVCs). Thus the drop in fVCs due to the oxoG filter suggests this sample tumor variants may have included oxoG artifacts.

Criteria STRAND builds on Criteria OXOG's filtered set by excluding variants deemed to be strand bias artifacts. UNMASC utilizes Rsamtools to count the number of forward or reverse strand and reference or alternate reads to conduct Fisher's Exact test per variant call. At a threshold of 0.05, variants with smaller p-values were considered strand bias artifacts. In Figure S10, the decrease in variability of PPV and mean increase in PPV appears to be attributed to strand bias artifacts present only among the TO variants and not the TMN variants. At last, Criteria FFPE utilized all previous criteria and filtered out variants deemed formalin-fixed paraffin-embedded artifacts. Below, the highlighted sample's variants did not appear to harbor paraffin artifacts after applying the previous sequence of filters.

A more in-depth look at Figure S10 illustrates the performance of a single sample in terms of SENS, PPV, and F1 measure (F1M) as they vary with respect to the layered filtering criteria, number of UMNs, and proportion variant called threshold.

The sample's PPV plateaued early at about 4 UMNs due to the presence of highly private germline variants not identified by 1000 Genomes or ExAC. Two false positive variants were considered hard-to-map by normal VAF segmentation. After applying Criteria C's filtering, the PPV increased from about 45% to 80% when each variant was required to appear in at least 90% of UMNs. Of the remaining TO variants, our OXOG approach inferred three OXOG variants with tumor VAFs of 0.021, 0.029, and 0.027 present in the TMN calls. After excluding inferred OXOG variants from both TMN and TO calls, this sample did not appear to have gold standard variants presenting with strand bias but they were present among TO calls. And finally, based on our proposed FFPE approach, the previously mentioned three OXOG variants also appeared to be FFPE artifacts. And thus for this sample, sensitivity was maintained at 100%, and with at least one UMN, the PPV is approximately 80% with a total of twelve TO variants after all filters are applied.

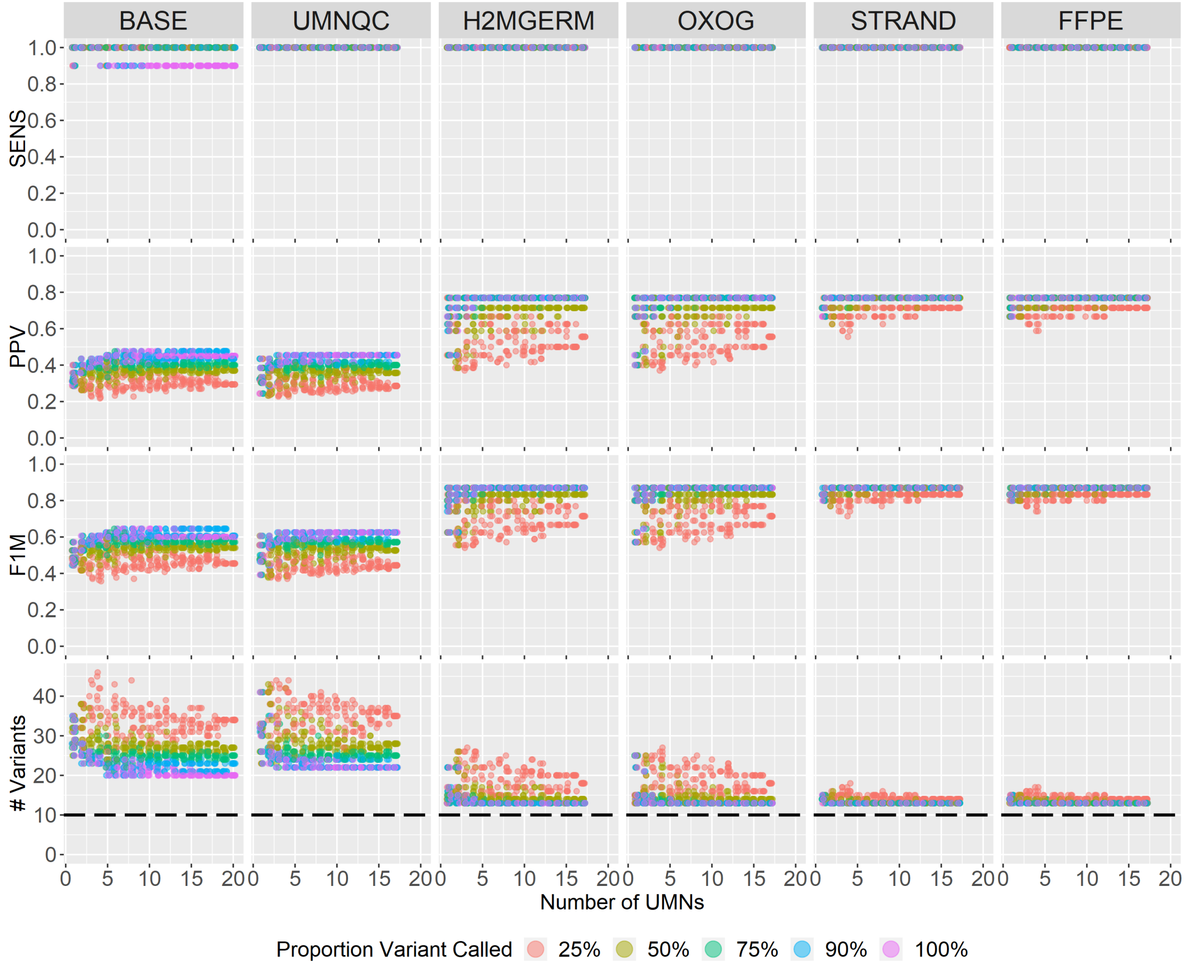

Figure S10 A summary of one sample's tumor-only variant calls. Columns correspond to the six cumulative filtering criterion. Rows correspond to sensitivity (SENS), positive predictive value (PPV), and number of filtered variant calls. The x-axis denotes the number of randomly selected unmatched normals to perform the variant calling. Colors denote the frequency threshold required to call a variant somatic. The dashed black line in the last row of plots denote the number of somatic variants from tumor with matched normal remaining after applying the filtering criterion.

5.1 Relating SENS and PPV to purity and mutation load

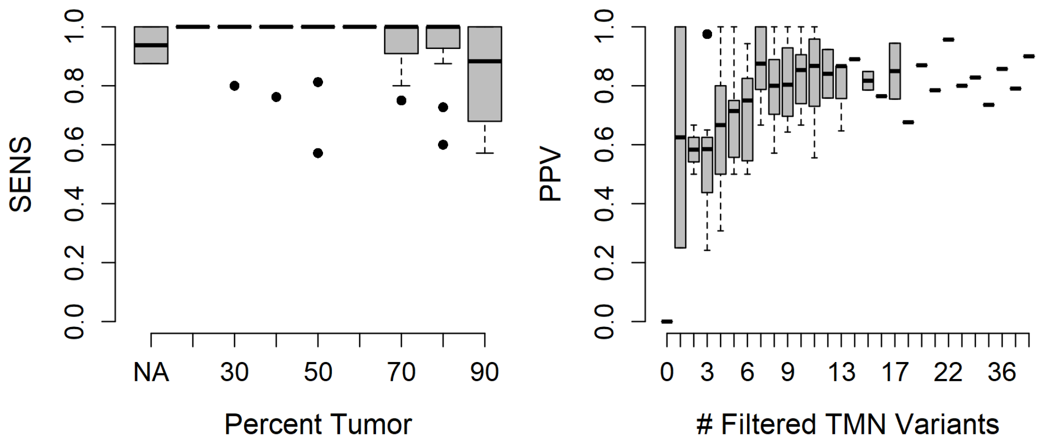

Figure S11 These plots assess the relationship between sensitivity and tumor purity in addition to positive predictive value and number of filtered tumor matched normal variant calls.

5.2 False Negatives

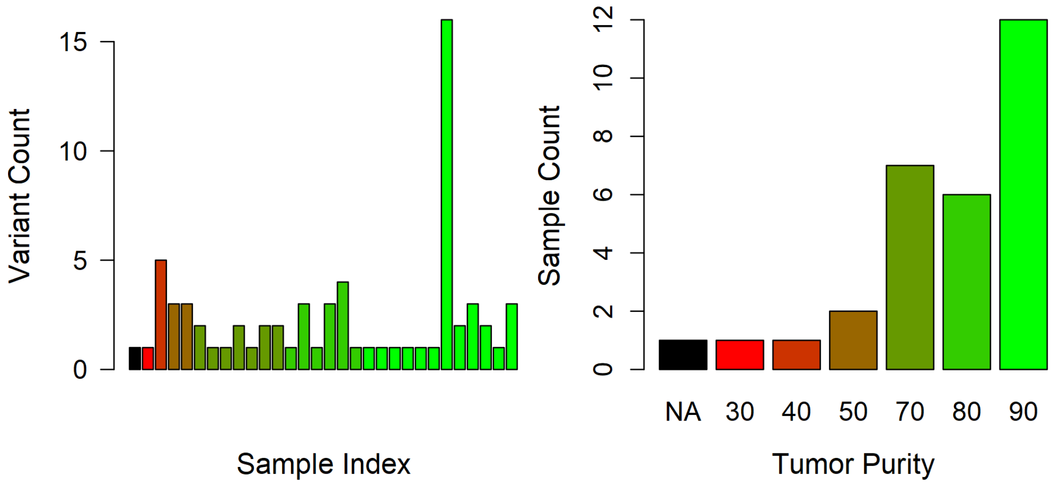

Figure S12 These plots capture the frequency of false negatives excluded by our workflow and the tumor purity of the 30 samples.

References

[1] Hiltemann,S., Jenster,G., Trapman,J., van der Spek,P. and Stubbs,A. (2015) Discriminating somatic and germline mutations in tumor DNA samples without matching normals. *Genome research.* **25**, 1382–1390.

[2] Smith,K.S., Yadav,V.K., Pei,S., Pollyea,D.A., Jordan,C.T. and De,S. (2016) SomVarIUS: somatic variant identification from unpaired tissue samples. *Bioinformatics.* **32**, 808–813.

[3] Sun,J.X., He,Y., Sanford,E., Montesion,M., Frampton,G.M., Vignot,S., Soria,J., Ross,J.S., Miller,V.A., Stephens,P.J. *et al.* (2018) A computational approach to distinguish somatic vs. germline origin of genomic alterations from deep sequencing of cancer specimens without a matched normal. *PLoS computational biology.* **14**, e1005965.

[4] Kalatskaya,I., Trinh,Q.M., Spears,M., McPherson,J.D., Bartlett,J.M. and Stein,L. (2017) ISOWN: accurate somatic mutation identification in the absence of normal tissue controls. *Genome medicine.* **9**, 1–18.

[5] Halperin,R.F., Carpten,J.D., Manojlovic,Z., Aldrich,J., Keats,J., Byron,S., Liang,W.S., Russell,M., Enriquez,D., Claasen,A. *et al.* (2017) A method to reduce ancestry related germline false positives in tumor only somatic variant calling. *BMC medical genomics.* **10**, 1–17.

[6] Halperin,R.F., Liang,W.S., Kulkarni,S., Tassone,E.E., Adkins,J., Enriquez, D., Tran,N.L., Hank,N.C., Newell,J., Kodira,C. *et al.* (2019) Leveraging spatial variation in tumor purity for improved somatic variant calling of archival tumor only samples. *Frontiers Oncology.* **9**, 119.

[7] Hsu,Y., Hsiao,Y., Kao,T., Chang,J. and Shieh,G.S. (2017) Detection of somatic mutations in exome sequencing of tumor-only samples. *Scientific reports.* **7**, 1–9.

[8] Madubata,C.J., Roshan-Ghias,A., Chu,T., Resnick,S., Zhao,J., Arnes,L., Wang,J. and Rabadan,R. (2017) Identification of potentially oncogenic alterations from tumor-only samples reveals Fanconi anemia pathway mutations in bladder carcinomas. *NPJ genomic medicine.* **2**, 1–10.

[9] Teer,J.K., Zhang,Y., Chen,L., Welsh,E.A., Cress,W.D., Eschrich,S.A. and Berglund,A.E. (2017) Evaluating somatic tumor mutation detection without matched normal samples. *Human genomics.* **11**, 1–13.

[10] Olshen,A.B., Venkatraman,E., Lucito,R. and Wigler,M. (2004) Circular binary segmentation for the analysis of array-based DNA copy number data. *Biostatistics.* **5**, 557–572.

[11] Li,H., Handsaker,B., Wysoker,A., Fennell,T., Ruan,J., Homer,N., Marth,G., Abecasis,G. and Durbin,R. (2009) The sequence alignment/map format and SAMtools. *Bioinformatics.* **25**, 2078–2079.

[12] Morgan,M., Pag'es,H., Obenchain,V. and Hayden,N. (2016) Rsamtools: Binary alignment (BAM), FASTA, variant call (BCF), and tabix file import. *R package version.* **1**, 677–689.

[13] Wong,S.Q., Li,J., Tan,A.Y., Vedururu,R., Pang,J.B., Do,H., Ellul,J., Doig,K., Bell,A., McArthur,G.A. *et al.* (2014) Sequence artefacts in a prospective series of formalin-fixed tumours tested for mutations in hotspot regions by massively parallel sequencing. *BMC medical genomics.* **7**, 1–10.

[14] Do,H. and Dobrovic,A. (2015) Sequence artifacts in DNA from formalin-fixed tissues: causes and strategies for minimization. *Clinical chemistry.* **61**, 64–71.

[15] Costello,M., Pugh,T.J., Fennell,T.J., Stewart,C., Lichtenstein,L., Meldrim,J.C., Fostel,J.L., Friedrich,D.C., Perrin,D., Dionne,D. *et al.* (2013) Discovery and characterization of artifactual mutations in deep coverage targeted capture sequencing data due to oxidative DNA damage during sample preparation. *Nucleic acids research.* **41**, e67–e67.
